# Rationalizing Enhanced Affinity of Engineered T‐Cell Receptors in Cancer Immunotherapy Through Interaction Energy Calculations and Residue Correlation Analysis

**DOI:** 10.1002/prot.70028

**Published:** 2025-08-01

**Authors:** Mario Frezzini, Daniele Narzi

**Affiliations:** ^1^ Department of Information Engineering, Computer Science and Mathematics University of L'aquila L'Aquila Italy; ^2^ Department of Physical and Chemical Sciences University of L'aquila L'Aquila Italy

**Keywords:** adoptive T‐cell therapy, allosteric pathways, binding affinity, major histocompatibility complex, molecular dynamics simulations, T‐cell receptor

## Abstract

The advancement of T cell engineering has significantly transformed the field of cancer immunotherapy. In particular, T cells equipped with modified T cell receptors present a promising therapeutic strategy, especially for addressing solid tumors. Nonetheless, critical obstacles, including suboptimal clinical response rates, off‐target toxicity, and the immunosuppressive nature of the tumor microenvironment, have impeded the full clinical implementation of this approach. Understanding the molecular determinants governing the interaction between T‐cell receptors and major histocompatibility complex molecules is pivotal not only for designing TCRs capable of selectively and effectively recognizing MHC on cancer cells but also for minimizing off‐target toxicity, thereby improving the safety profile of TCR‐based therapies. In this study, we used a test case involving a natural TCR (c728) and its affinity‐enhanced variant (c796), which differ by a single conservative mutation in the βCDR1 region. Through molecular dynamics simulations, MM/PBSA binding energy and Free Energy Perturbation calculations, residue‐specific energy decomposition, and correlation analyses, we dissected the molecular basis of the engineered TCR's six‐fold increase in binding affinity for the peptide–MHC complex compared to its parental counterpart. Interestingly, our results indicate that this affinity enhancement is not directly attributable to the mutation itself but rather to the dynamic interplay of both proximal and distal residues that are either directly correlated with the mutation or connected via allosteric pathways. Our findings, which align with experimental data, highlight the nuanced role of structural flexibility and allosteric communication in shaping TCR‐pMHC interactions. By demonstrating the utility of combining computational techniques to unravel these dynamics, this work emphasizes how similar approaches can guide the rational design of engineered TCRs with improved efficacy and specificity, advancing their application in cancer immunotherapy.

## 1 Introduction

Antigen recognition by T cells plays a fundamental role in the adaptive immune response in humans. T cells are pivotal in identifying and eliminating infected or malignant cells, a process that relies on the specificity of T‐cell receptors (TCRs) [1]. These highly specialized receptors recognize antigens presented by major histocompatibility complex (MHC) molecules on the surface of antigen‐presenting cells. TCRs bind to peptide‐MHC (pMHC) complexes with remarkable precision, enabling the immune system to distinguish between self and non‐self peptides.

The major histocompatibility complex, referred to as the human leukocyte antigen (HLA) in humans, and the T‐cell receptor form an essential interface within the immune system. This interaction involves the alpha helices of the MHC binding groove, which present antigenic peptides, and the variable loops of the TCR, which are specifically adapted to recognize these peptides [2]. The complementary determining regions (CDR) of the TCR engage precisely with the peptide presented by the MHC, facilitating the recognition of target cells. This essential interaction, dictated by distinct structural conformations, not only governs immune responses but also underpins the development of immunotherapy‐based treatments.

In particular, the adoptive transfer of effector T cells engineered to target tumor‐associated antigens represents a promising strategy in cancer immunotherapy [3]. However, the clinical application of naturally occurring tumor‐reactive T cells faces significant challenges. Thymic selection eliminates T cells with high affinity for self‐antigens, while the low abundance of peptide‐major histocompatibility antigen complexes on some tumor cells further compromises therapeutic efficacy.

To address these challenges, engineering tumor‐specific TCRs with enhanced affinity has emerged as a strategy to improve tumor cell recognition and killing. This approach, optimizing TCR affinity within the physiological range, has demonstrated enhanced clinical efficacy compared to native TCRs [4, 5]. Affinity‐enhanced TCRs show promise in a broader range of cancer indications, surpassing the limited success observed with naturally occurring tumor‐infiltrating lymphocytes, especially in malignant melanoma.

Over the past three decades, extensive studies into the structural properties of TCR/pMHC complexes, primarily through x‐ray crystallography, have provided invaluable insights into the binding mechanisms of natural TCRs to antigens [2, 6, 7, 8, 9]. While these investigations have elucidated the detailed binding mode of TCR/pMHC complexes, fully understanding the mechanism of antigen recognition by TCRs requires consideration of additional factors. Notably, the conformational flexibility of the complex and the allosteric communication between its various regions play critical roles [10, 11, 12]. In this regard, computational approaches, such as molecular dynamics (MD) simulations, have emerged as powerful tools to explore the dynamic aspects of TCR‐pMHC interactions [11, 13, 14, 15, 16, 17, 18, 19, 20, 21, 22, 23, 24].

The rapid advancements in artificial intelligence (AI) over recent years offer significant potential in studying and designing engineered T‐cell receptors for cancer immunotherapy [25, 26, 27]. AI‐driven approaches can accelerate the identification of mutations that enhance the affinity of TCRs toward oncological targets, leveraging experimentally resolved or computationally modeled TCR/pMHC complex structures. However, strategies solely focused on optimizing binding affinity from static structural data often overlook the critical dynamic aspects of TCR recognition mechanisms. Our study leverages MD simulations to explore the dynamic behavior of TCR/pMHC complexes, providing a test case that underscores the potential of this approach in advancing TCR engineering.

**FIGURE 1:**
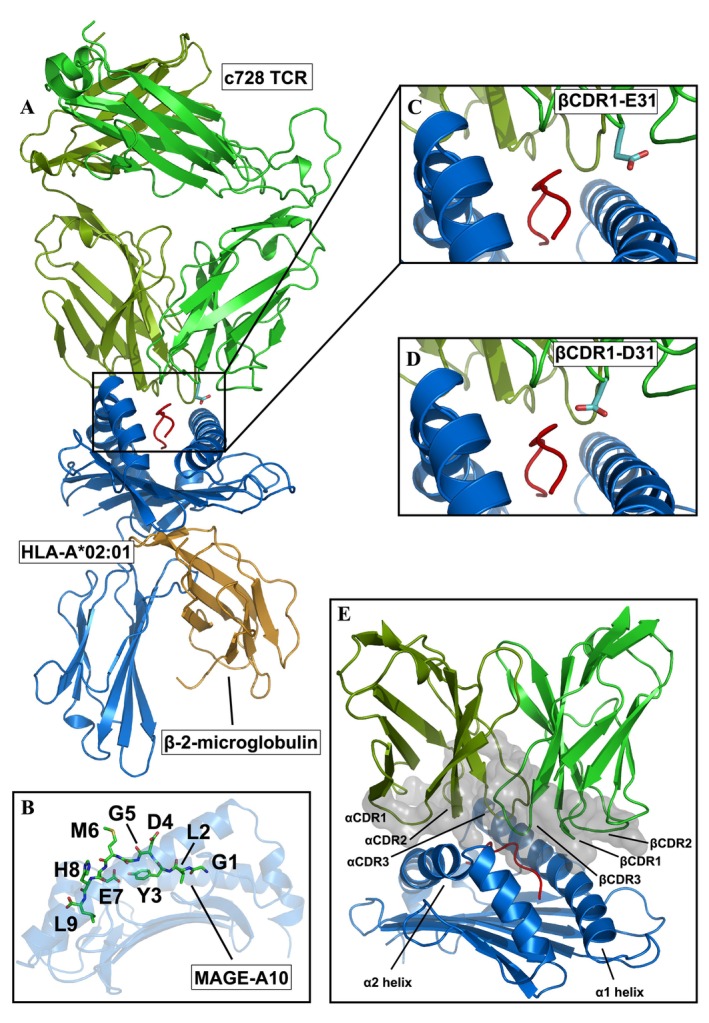
Structure of the c728 TCR–HLA‐A*02:01/MAGE‐A10 complex. (A) Cartoon representation of the c728 TCR–HLA‐A*02:01/MAGE‐A10 complex (PDB ID: 7PDW). (B) Stick representation of the MAGE‐A10 peptide bound to the binding groove of HLA‐A*02:01. (C) Close‐up view of the β CDR1‐E31 residue represented as sticks in the c728 TCR–HLA‐A*02:01/MAGE‐A10 complex. (D) Close‐up view of the β CDR1‐D31 residue represented as sticks in the c796 TCR–HLA‐A*02:01/MAGE‐A10 complex. (E) Cartoon representation of the HLA‐A*02:01/MAGE‐A10 complex bound to the c728 TCR, with the TCR loops interacting with the HLA‐A*02:01/MAGE‐A10 complex highlighted.

As a test case, we focused on an affinity‐enhanced TCR targeting a melanoma antigen gene MAGE‐A10 peptide presented by HLA‐A*02:01. This engineered TCR (referred to as c796 TCR) was recently developed starting from its parental TCR, c728 [28, 29]. The c796 TCR differs from its parental counterpart by a single conservative mutation in the β‐CDR1 loop, where glutamate (E31) is substituted with aspartate (D31) (Figure 1). Despite this minimal modification, experimental data obtained through surface plasmon resonance revealed a remarkable six‐fold increase in the binding affinity of c796 to the peptide–HLA (pHLA) complex compared to the parental TCR [28]. This case highlights the potential for seemingly minor changes in TCR sequence to produce significant effects on binding affinity, making it an ideal system to explore the dynamic and structural factors underpinning such enhancements. Importantly, both the parental and engineered TCR–pMHC complexes have been resolved experimentally at high resolution (PDB IDs: 7PBC and 7PDW), and show only minimal structural differences. This makes the system particularly well suited for a controlled computational analysis, where all observed effects can be directly linked to the single conservative mutation. As such, the c728/c796 pair provides an ideal benchmark for testing a simulation‐based methodology that we aim to generalize in future studies to other engineered TCRs.

In this study, we performed molecular dynamics simulations to investigate the structural and energetic differences between the parental (c728) and engineered (c796) TCRs in complex with the pMHC, as well as for the isolated TCRs and pMHC. Structural stability and flexibility of the two TCR/pMHC systems were assessed to identify distinct dynamic behaviors. We calculated the interaction energies between TCRs and pMHC using both Free Energy Perturbation and MM/PBSA calculations, assessing the impact of dynamics on binding affinity and identifying the contribution of individual residues to the observed differences in binding affinity. This analysis followed a scheme similar to that employed by Ozbek and coworkers [30]. Furthermore, correlation analyses were conducted to explore the relationships between the mutated residue (β CDR1‐E31/D31) and other key residues in the TCR. Finally, we identified potential allosteric pathways linking these residues, shedding light on the dynamic mechanisms underlying the enhanced affinity of the engineered TCR.

Several studies have previously employed molecular dynamics simulations and advanced analytical techniques to investigate the structural and dynamic properties of TCR/pMHC complexes in the context of immunotherapy and/or engineered TCRs [31, 32, 33, 34, 35, 36, 37]. This study stands out for employing a dynamic approach to investigate the interplay between conformational flexibility, binding affinity, and allosteric communication in an engineered TCR compared to its parental counterpart, specifically in the context of cancer immunotherapy with the broader aim of establishing a generalizable computational strategy for the analysis of engineered TCRs. Integrating this methodology with AI‐driven strategies to predict and propose targeted mutations could further enhance the rational design of next‐generation TCRs with improved specificity and efficacy for tumor‐associated antigens, ultimately advancing cancer immunotherapy.

## 2 Results

In this work, we carried out classical MD simulations of the two complexes formed by human leucocyte antigen HLA‐A*02:01 presenting the MAGE‐A10 9‐mer peptide with the c728 TCR receptor (PDB ID: 7PDW) and the c796 TCR receptor (PDB ID: 7PBC), respectively [29]. Additionally, to assess the effect of the mutation on TCR and pMHC flexibility in both bound and unbound states, we also simulated the isolated HLA‐A*02:01 presenting the peptide MAGE‐A10 and the two receptors c728 TCR and c796 TCR. These simulations serve as a reference to comparatively evaluate how the mutation impacts the conformational dynamics of each component, independently in the bound and unbound conditions. In total, five different systems were simulated, as reported in Table 1. For each system, three independent trajectories of one microsecond each were simulated.

**TABLE 1:** List of simulated systems.

Simulated system	System size (atoms)	Time length	Number of replicas
c728 TCR–HLA‐A*02:01/MAGE‐A10	311 406	1000 ns/500 ns	3/5 (8 independent replicas)
c796 TCR–HLA‐A*02:01/MAGE‐A10	297 825	1000 ns/500 ns	3/5 (8 independent replicas)
c728 TCR	76 168	1000 ns	3 independent replicas
c796 TCR	73 722	1000 ns	3 independent replicas
HLA‐A*02:01/MAGE‐A10	76 410	1000 ns	3 independent replicas

*Note*: The total number of atoms present in each simulated system, including protein, solvent, and ions is reported, as well as the time length of a single trajectory and the number of independent replicas carried out for each system.

The simulations were conducted at constant pressure and temperature (i.e., P=1atm, T=310K in a 150mM NaCl solution). The analyses performed on the various trajectories, where they refer to time‐averaged values, were carried out neglecting the first 200ns of each trajectory and averaging over the remaining 800 ns across the three replicas. Thus, for each simulated system, the average values obtained were derived from a sampling of 2.4μs for each simulated system. Throughout the text, any specific reference to one of the three replicas will be indicated as R1, R2, or R3.

In this section, we present the analyses aimed to show the overall stability of the various simulated systems and the structural and dynamic differences observed among them. We will then show the calculations of TCR‐pMHC interaction energy for the crystallographic structures, comparing them with those obtained by averaging over the simulated trajectories to quantify both the effect of the mutation in TCR c796 and the impact of dynamics on the TCR‐pMHC binding energy. Finally, we will identify the residues that contribute most to the differences in interaction energy between the HLA‐A*02:01/MAGE‐A10‐9 complex and the two investigated TCR, highlighting any correlations between these residues and the amino acid subject to mutation.

### 2.1 Overall System Stability

The overall stability of the simulated protein systems reported in Table 1 was verified by monitoring the time evolution of different properties along the respective trajectories (see Figures S1–S3). In particular, the Root Mean Square Deviation (RMSD) trend shows that the structures of both the two TCR/pMHC complexes (Figure S1A) and the individually simulated TCR c728, TCR c796, and HLA‐A*02:01/MAGE‐A10‐9 systems (Figures S2A and S3A) stabilize after 200ns. For this reason, the average values of structural properties and interaction energies shown below in the text were obtained neglecting the first 200ns of the MD trajectories. The radius of gyration as a function of time (Figures S1B, S2B, and S3B) generally show values similar to or slightly greater than those calculated from the x‐ray structure. In no case does the radius of gyration show an increasing trend, which would indicate potential deterioration of the tertiary and quaternary structure of the simulated complexes. Similarly, the number of H‐bonds within the different protein systems oscillates around an average value similar to that found in the crystallographic structure in the case of the individually simulated TCR c728 and TCR c796 systems (Figure S2C) and slightly lower in the case of the two TCR/pMHC complexes (Figure S1C) and the individually simulated HLA‐A*02:01/MAGE‐A10‐9 complex (Figure S3C), thus indicating a substantial preservation of the secondary structures.

**FIGURE 2:**
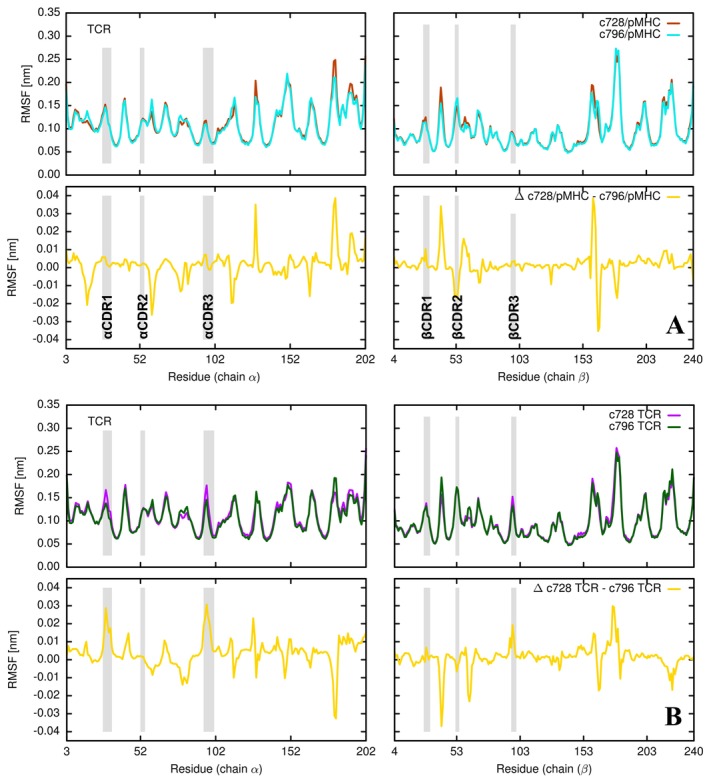
Root mean square fluctuations of protein residues (A) RMSF of the amino acid backbone in chain α (left) and chain β (right) of the c728 TCR (red trace) and c796 TCR (cyan trace), calculated over the trajectories of the TCR/pMHC complexes. The difference in RMSF between the two TCRs is shown as a yellow trace. TCR loops interacting with the HLA‐A*02:01/MAGE‐A10 complex are highlighted in gray. (B) RMSF of the amino acid backbone in chain α (left) and chain β (right) of the c728 TCR (magenta trace) and c796 TCR (green trace), calculated over the trajectories of the isolated TCRs. The difference in RMSF between the two TCRs is shown as a yellow trace. All RMSF values were block‐averaged over 100 ns time windows, excluding the first 200 ns of each trajectory.

In order to monitor the flexibility of the various regions of the two simulated TCR/pMHC complexes, as well as the two TCR receptors and the HLA‐A*02:01/MAGE‐A10 complex simulated individually, we calculated the respective root mean square fluctuations (RMSF) per residue. The RMSFs reported in Figure 2A show the different fluctuations between TCR c728 and TCR c796 when simulated bound to the HLA‐A*02:01/MAGE‐A10 complex. Our simulations do not show significant differences in the mobility of the α CDR1‐3 loops in the two receptors, with only a slight increase in the β CDR2 loop in the TCR c796 receptor compared to the parental TCR c728. When looking at the RMSF per residue in the two receptors simulated individually (Figure 2B), a greater flexibility of the α CDR1 and α CDR3 loops is observed in the TCR c728 receptor compared to the engineered TCR c796 receptor. Only minimal differences are observed in the fluctuations of the β CDR1‐3 loops. When comparing separately the fluctuations present in the two receptors simulated in the TCR/pMHC complex with the fluctuations present in the simulations of the two isolated receptors (Figure S4), no significant differences are observed between the two receptors regarding the mobility of the α CDR1‐3 and β CDR1‐3 loops.

The RMSF of the binding groove of the HLA‐A*02:01/MAGE‐A10 complex when bound to the TCR c728 receptors shows an increase in flexibility of the C‐terminal region of the α1 helix, located near the β CDR1‐3 loops (see also Figure 1E), compared to the respective HLA‐A*02:01/MAGE‐A10 complex bound to TCR c796 (Figure S5). The α2 helix, on the other hand, does not show significant differences in mobility between the two complexes. This trend is also clear when we examine the differences in flexibility of the HLA‐A*02:01/MAGE‐A10 binding groove between the two TCR/pMHC complexes and the HLA‐A*02:01/MAGE‐A10 complex simulated individually (Figure S5).

The fluctuations of the MAGE‐A10 peptide side chains when bound to MHC alone and in the TCR/pMHC complexes were analyzed, and the respective RMSF values are reported in Figure S6. As expected, our analyses show more pronounced fluctuations of MAGE‐A10 residues when bound to MHC alone compared to the TCR/pMHC complex. On the other hand, the E31D mutation does not appear to have a significant impact on the mobility of MAGE‐A10 amino acids, as the difference in RMSF values of these residues between the two simulated TCR/pMHC systems is approximately zero across the entire MAGE‐A10 peptide.

### 2.2 TCR–pMHC Interaction Energy

The incorporation of a single conservative mutation into the c728 TCR was experimentally found to result in a six‐fold increase in the binding affinity of the engineered c796 TCR for the HLA‐A*02:01/MAGE‐A10 complex [28].

In this regard, we performed binding energy calculations between the two investigated TCRs and the HLA‐A*02:01/MAGE‐A10 complex to assess the influence of dynamics on the TCR‐MHC binding affinity compared to the affinity calculated based on the single crystallographic structure. The simplest, though more approximate, way to estimate the difference in binding affinity between the two receptors considered in this study is to consider only the contributions from short‐range (SR) Coulomb and Lennard‐Jones (LJ) interactions, applying a cut‐off beyond which interactions are considered null.

Regarding the crystallographic structure alone, our calculations, reported in Table 2, indicate that by considering only the SR contributions, the complex between the engineered c796 TCR and the HLA‐A*02:01/MAGE‐A10 complex is 15 kJ/mol less stable than the respective system with the parental TCR. This result, therefore, contradicts the experimental evidence reported in Reference [28]. On the other hand, the calculation of the polar solvation energy shows a 6kJ/mol higher affinity for the system with the engineered TCR, while the contribution of the apolar solvation energy is approximately equal in both systems.

**TABLE 2:** TCR‐pMHC binding energy calculated from the x‐ray structure.

	c728 TCR–HLA‐A*02:01/MAGE‐A10	c796 TCR–HLA‐A*02:01/MAGE‐A10
$\Delta E_{\mathrm{SR}}$	−992.2	−977.2
$\Delta E_{\text{Coul}/\mathrm{LJ}}$	−1568.7	−1623.7
$\Delta E_{\text{solv}-\text{polar}}$	696.1	690.1
$\Delta E_{\text{solv}-\text{apolar}}$	−55.7	−55.0

*Note*: Values are reported in kJ/mol. For further details, refer to Section Energy calculations.

In total, considering SR interactions for the Coulomb and LJ energy contributions in the crystallographic structure leads to binding energy values that erroneously suggest a lower binding affinity of the engineered TCR for the HLA‐A*02:01/MAGE‐A10 complex compared to the parental TCR. However, if instead of considering only the SR interactions, we also include the energy contributions accounting for interactions beyond the cut‐off distance, the results show a qualitatively opposite trend, consistent with the experimental evidence. In this case, the complex with the engineered TCR, with respect to the Coulomb and LJ energetic contributions ($\Delta E_{\text{Coul}/\mathrm{LJ}}$), is 55kJ/mol more stable than the complex with the parental TCR.

The same energy calculations performed on the crystallographic structures were repeated on the molecular dynamics trajectories, obtaining average values while disregarding the first 200 ns of each simulation. The values reported in Table 3 show that, unlike the respective values obtained from the crystallographic structures, the sum of the Coulomb and LJ short‐range contributions indicates greater stability of the TCR/MHC complex in the case of the engineered TCR. More precisely, the energy difference between the two systems (${\left\langle \Delta E_{\mathrm{SR}}\right\rangle }_{\mathrm{MD}}$) is 150kJ/mol. However, when we also consider the contribution of polar solvation energy, we observe that, in this case, the engineered TCR/MHC complex is about 110kJ/mol less stable than the system with the parental TCR. The contribution of apolar solvation energy remains similar in both cases studied.

**TABLE 3:** TCR‐pMHC binding energy averaged over three 1000ns‐long simulated trajectories.

	c728 TCR–HLA‐A*02:01/MAGE‐A10	c796 TCR–HLA‐A*02:01/MAGE‐A10
${\left\langle \Delta E_{\mathrm{SR}}\right\rangle }_{\mathrm{MD}}$ (${\left\langle \sigma_{\mu }\right\rangle }_{\mathrm{MD}}$)	−746.2 (10.1)	−896.2 (26.4)
${\left\langle \Delta E_{\text{Coul}/\mathrm{LJ}}\right\rangle }_{\mathrm{MD}}$ (${\left\langle \sigma_{\mu }\right\rangle }_{\mathrm{MD}}$)	−941.7 (30.2)	−1229.6 (51.0)
${\left\langle \Delta E_{\text{solv}-\text{polar}}\right\rangle }_{\mathrm{MD}}$ (${\left\langle \sigma_{\mu }\right\rangle }_{\mathrm{MD}}$)	490.6 (9.3)	599.7 (21.2)
${\left\langle \Delta E_{\text{solv}-\text{apolar}}\right\rangle }_{\mathrm{MD}}$ (${\left\langle \sigma_{\mu }\right\rangle }_{\mathrm{MD}}$)	−49.0 (0.5)	−51.9 (0.7)

*Note*: Standard deviations of the mean are provided in parentheses. Values are reported in kJ/mol. For further details, refer to Section Energy calculations.

In conclusion, considering all the energy contributions averaged over the various simulations performed, excluding the entropic contribution, the engineered c796 receptor is globally more affine to the HLA‐A*02:01/MAGE‐A10 complex than the parental TCR. This is qualitatively in agreement with the experimental evidence [28]. However, it should be noted that, in principle, differences in entropic contributions between the parental and engineered TCR binding processes should also be taken into account. This contribution is difficult to estimate quantitatively due to limited sampling issues (see also Reference [38]). Nevertheless, we attempted a preliminary evaluation of the relative entropic effects by computing Schlitter entropy [39] estimates from the 1μs trajectories for both the isolated TCRs and the corresponding TCR–pMHC complexes (parental and engineered). The resulting contributions, reported in Table S1, indicate that the entropic term also favors binding of the engineered TCR to the pMHC compared to the parental receptor $\left(-T{\mathrm{\Delta \Delta S}}_{b}=-18.6\;\mathrm{kJ}/\mathrm{mol}\right)$. We emphasize, though, that these results should be interpreted with caution, as achieving sufficient phase space sampling to ensure convergence of entropy estimates remains unfeasible for systems of this size.

When we also account for energy contributions beyond the cut‐off distance, the situation remains qualitatively similar to the case of short‐range contributions alone, but quantitatively the difference becomes even more significant. The difference in the energetic contribution ${\left\langle \Delta E_{\text{Coul}/\mathrm{LJ}}\right\rangle }_{\mathrm{MD}}$ between the two systems is now approximately 288kJ/mol, significantly greater in absolute value than the difference in polar solvation energy contributions between the two systems.

Being aware that the large value obtained clearly overestimates the experimentally observed six‐fold increase in affinity, we repeated the MM/PBSA calculation on a larger set of MD simulations to assess whether varying the number of independent simulations, while keeping the total sampling time identical to the previous analysis, would substantially affect the results. Specifically, we performed ten additional 500 ns MD simulations (five per TCR/pMHC complex) and repeated the MM/PBSA analysis using eight simulations per system (the five new trajectories plus the three 1 μs trajectories mentioned above), analyzing the time window from 200 to 500 ns for each trajectory, with a total sampling of 2.4μs per system, identical to that of the previous analysis. As reported in Table S2, this new calculation showed a substantial reduction in the binding energy gap between the parental and engineered TCRs, with the engineered TCR now estimated to bind more favorably by 39.5 kJ/mol compared to the 288 kJ/mol difference previously calculated.

**FIGURE 3:**
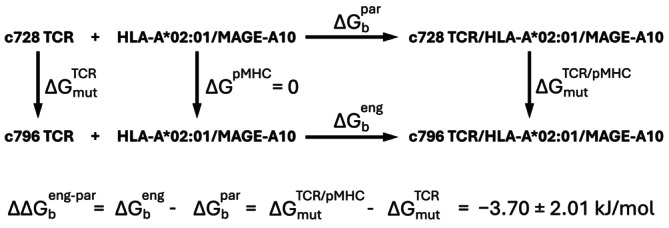
Thermodynamic cycle for FEP calculations. The relative binding free energy ($\Delta \Delta G_{b}^{\mathrm{eng}-\mathrm{par}}$) between the engineered TCR c796 and the parental TCR c728, both binding the same pMHC, is calculated as the difference between $\Delta G_{\mathrm{mut}}^{\mathrm{TCR}/\text{pMHC}}$ and $\Delta G_{\mathrm{mut}}^{\mathrm{TCR}}$. These correspond to the free energy changes associated with the alchemical transformation from Glu to Asp in the TCR/pMHC complex and in the isolated TCR, respectively.

An alternative to MM/PBSA for evaluating differences in binding affinity between engineered and parental TCRs is provided by free energy perturbation (FEP) calculations. However, FEP can be extremely challenging in terms of convergence and computational cost for systems of this size. Nonetheless, previous studies have successfully applied FEP to TCR/pMHC binding systems [40, 41]. To test the practicality of this approach on our system, we performed FEP calculations following the protocol suggested by de Groot group [42]. Technical details are reported in the Methods section. Our FEP analyses, shown in Figure S7, indicate a slightly more favorable binding free energy for the engineered TCR compared to the parental receptor ($\Delta \Delta G_{b}^{\mathrm{eng}-\mathrm{par}}=-3.7\pm 2.01$ kJ/mol, see also Figure 3). While this result is qualitatively consistent with experimental expectations, the relative uncertainty of this estimate (54%) requires appropriate caution in its interpretation, highlighting the challenges of achieving robust convergence in such large macromolecular systems.

### 2.3 Pairwise Interaction Energies Between TCR and pMHC Residues

In order to identify which residues contribute the most to the binding energy differences between the two TCRs investigated and the pMHC complex, we calculated the Pairwise Interaction Energies between the residues of the TCRs and the residues of the pMHC complex, as explained in the Materials and methods section (see also Figure S8).

**FIGURE 4:**
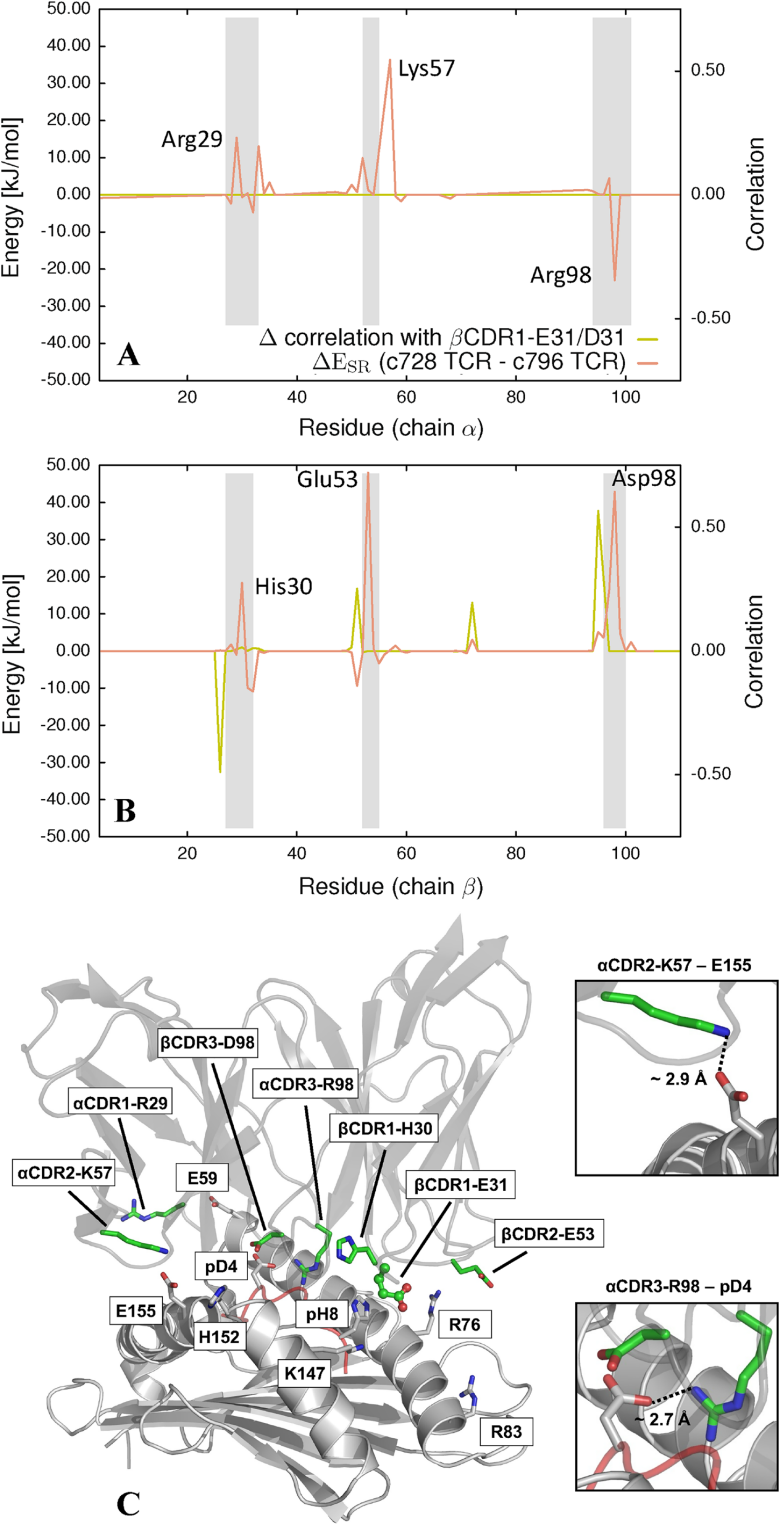
Interaction energy differences of TCR residues with pMHC. (A) Differences in interaction energies ($\Delta E_{\mathrm{SR}}$) for selected residues of the α chain of the TCRs with pMHC between the two investigated TCRs. (B) Differences in interaction energies ($\Delta E_{\mathrm{SR}}$) for selected residues of the β chain of the TCRs with pMHC between the two investigated TCRs. The difference in correlation between residues of the c728 TCR and the E31 residue, compared to the correlation between residues of the c796 TCR and the D31 residue, is also shown as a yellow line. (C) Stick representation of selected TCR residues exhibiting the largest interaction energy differences with pMHC residues between the two investigated TCRs, along with the corresponding pMHC residues identified as interacting partners in the x‐ray structure and/or along the simulated trajectories.

In Figure 4A,B, we report the interaction energy differences between the two simulated systems, relative to the TCR residues that, in at least one frame of the MD simulations, are within 6Å of any residue of the two α‐helices and the MAGE‐A10 peptide of the HLA‐A*02:01/MAGE‐A10 complex. These residues are highlighted in Figure 4C, along with the interacting residues of the pMHC complex. Our analysis shows that in the α chain of the engineered TCR c796, residues α CDR1‐R29 and α CDR2‐K57 exhibit interaction energies (in absolute value) with the pMHC complex residues greater compared to those of the parental TCR c728. In contrast, residue α CDR3‐R98 shows a stronger interaction with the pMHC complex residues in the parental TCR c728 compared to the engineered TCR. β CDR1‐H30, β CDR2‐E53, and β CDR3‐D98 all exhibit interaction energy values with the pMHC complex residues in absolute terms greater than the parental TCR c728. It is interesting to note that the β CDR1‐E31/D31 residue, which is the subject of mutation, does not show a significant difference in interaction energy with the pMHC complex residues in the two studied systems.

We then analyzed the correlation between the β CDR1‐E31/D31 residue and other TCR residues along the simulated trajectories, as explained in the Materials and methods section. As expected, we did not find significant correlations between this residue and the other TCR residues in the α chain. In the β chain, however, the correlations are very evident in both simulated systems, particularly with the TCR residues located near the three loops β CDR1, β CDR2, and β CDR3 (see Figure S9). We reported the difference in correlation between the two investigated systems in Figure 4A,B. It is interesting to note that the largest differences in correlation between the two systems occur near the residues of the TCR β chain that are characterized by a greater difference in interaction energy with the pMHC complex residues.

In order to structurally understand the origin of the different interaction energies between TCR residues and pMHC complex residues in the investigated systems, we analyzed the behavior of the respective residue‐to‐residue distances sampled along the MD simulations.

**FIGURE 5:**
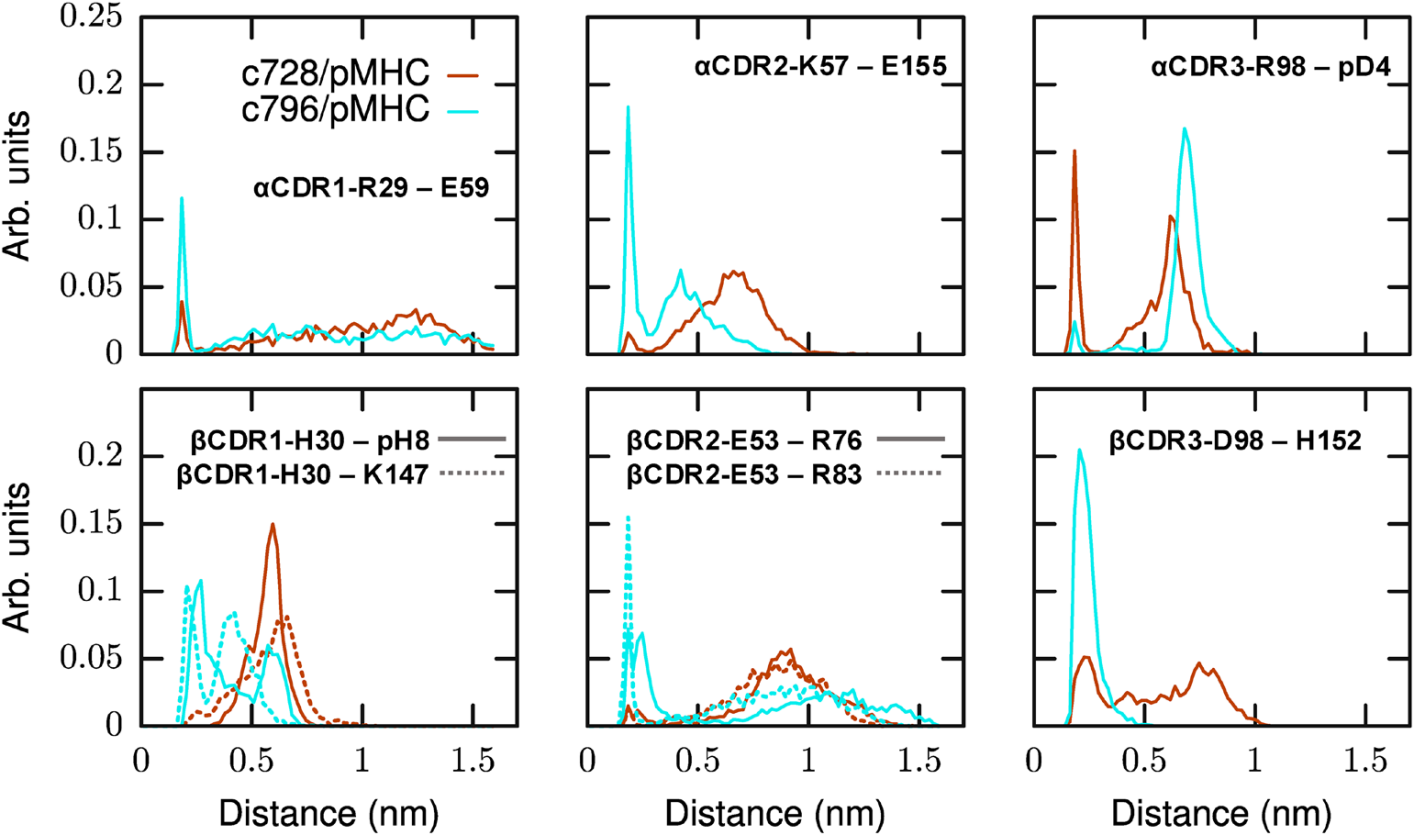
Distribution of selected distances within the TCR/MHC complexes. Top left panel: Distance between α CDR1‐R29 and E59; Top middle panel: Distance between α CDR2‐K57 and E155; Top right panel: Distance between α CDR3‐R98 and pD4; Bottom left panel: Distances between β CDR1‐H30 and K147, and between β CDR1‐H30 and pH 8; Bottom middle panel: Distances between β CDR2‐E53 and R76, and between β CDR2‐E53 and R83; Bottom right panel: Distance between β CDR3‐D98 and H152. Distance distributions calculated for c728 residues interacting with pMHC are shown in red, while those for c796 residues interacting with pMHC are shown in cyan. All distributions were computed by averaging over three replicas of each system, neglecting the first 200ns of each trajectory.

**FIGURE 6:**
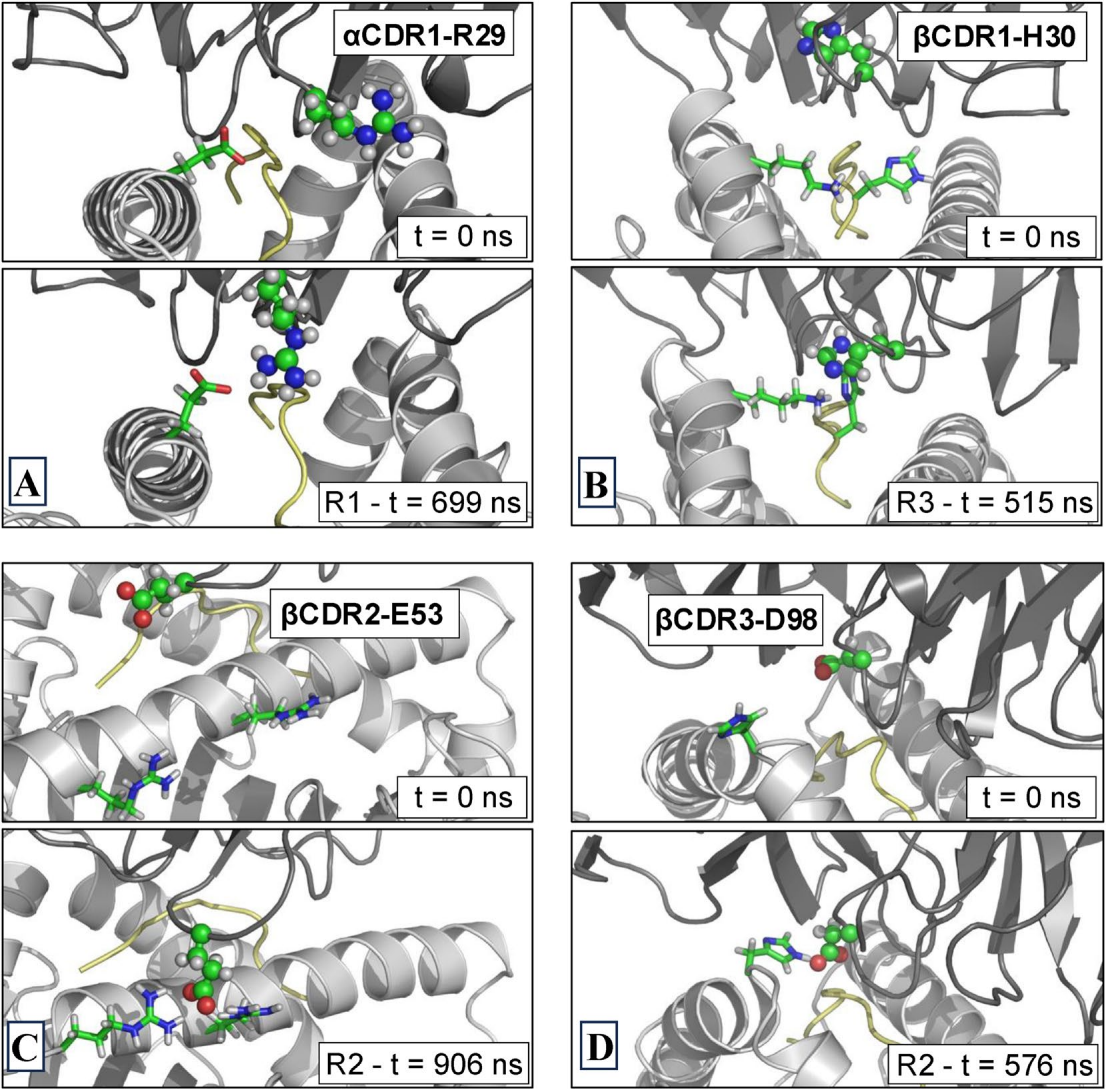
Representative snapshots from MD trajectories of the c796 TCR–HLA‐A*02:01/MAGE‐A10 complex. (A) Conformation of α CDR1‐R29 (depicted as ball‐and‐stick) at t=0ns and t=699ns from the R1 simulation. (B) Conformation of β CDR1‐H30 (depicted as ball‐and‐stick) at t=0ns and t=515ns from the R3 simulation. (C) Conformation of β CDR2‐E53 (depicted as ball‐and‐stick) at t=0ns and t=906ns from the R2 simulation. (D) Conformation of β CDR3‐D98 (depicted as ball‐and‐stick) at t=0ns and t=576ns from the R2 simulation.

The distribution of these distances is shown in Figure 5. The corresponding contact occupancy and stability data are reported in Table S3. Regarding the TCR α chain, there are two pairs of residues, namely α CDR2‐K57–E155 and α CDR3‐R98–pD4, which interact via salt bridges in both crystallographic structures (see also Figure 4). However, these interactions are partially lost during the simulated trajectories. More specifically, the α CDR2‐K57–E155 interaction is more persistent in the system with the engineered TCR compared to the parental TCR, whereas the α CDR3‐R98–pD4 interaction shows the opposite behavior, being more persistent in the system with the parental TCR than in the engineered TCR system. These trends explain the differences in interaction energy reported in Figure 4 concerning the α CDR2‐K57 and α CDR3 residues. Regarding the α CDR1‐R29 residue, there is no strong interaction between this residue and any residue of the pMHC complex in the crystallographic structure for both systems. However, during the molecular dynamics simulations, a transient salt bridge forms between the α CDR1‐R29 residue and the E59 residue, which is more persistent in the system with the engineered TCR compared to the system with the parental TCR (see also the top left panel of Figure 6).

Moving to the TCR β chain, two of the three residues that show the greatest differences in interaction energies with the pMHC complex between the two studied systems (i.e., β CDR1‐H30 and β CDR2‐E53) are not in close contact with any residue of the pMHC complex in their crystallographic positions. However, during the simulated trajectories, various contacts are formed between these residues and some residues of the pMHC complex. More specifically, the β CDR1‐H30 residue forms contacts with residues K147 and pH 8, the latter belonging to the MAGE‐10 peptide (see the top right panel of Figure 6). The β CDR2‐E53 residue forms contacts with residues R76 and R83 located on the α1 helix of HLA‐A*02:01 (see the bottom left panel of Figure 6). From the analysis of the distances between the aforementioned residues reported in Figure 5, it is clear that the residue‐to‐residue distances are, on average, shorter in the system with the engineered TCR than in the system with the parental TCR. Finally, regarding the β CDR3‐D98 residue, an H‐bond is present between this residue and the Q156 residue (located on the α2 helix of HLA‐A*02:01) in both crystallographic structures of the two systems investigated here. This interaction remains relatively stable during the molecular dynamics simulations of both systems (data not shown). However, in the case of the system with the engineered TCR, the β CDR3‐D98 residue stably interacts also with the H152 residue via H‐bond (see the bottom right panel of Figure 6), whereas this interaction is only partially present in the parental TCR system (see Figure 5).

In conclusion, the set of distances between TCR residues and pMHC complex residues sampled along the simulated trajectories is consistent with the respective differences in interaction energies reported in Figure 4.

### 2.4 Preferential Allosteric Pathways Starting From β CDR1‐E31/D31

As expected, and shown in Figure S8, we identified a direct correlation between β CDR1‐E31/D31 and β CDR1‐H30. In contrast, the other five residues highlighted in Figure 4C (α CDR1‐R29, α CDR2‐K57, α CDR3‐R98, β CDR2‐E53, and β CDR3‐D98) do not exhibit direct correlations with β CDR1‐E31/D31. In order to clarify the mechanistic basis by which the *β* CDR1‐E31D mutation could influence, through allosteric pathways, residues that significantly contribute to the binding energy difference between the two TCRs, we analyzed the preferential allosteric paths connecting the mutation site to the key energetic contributors identified in our study.

**FIGURE 7:**
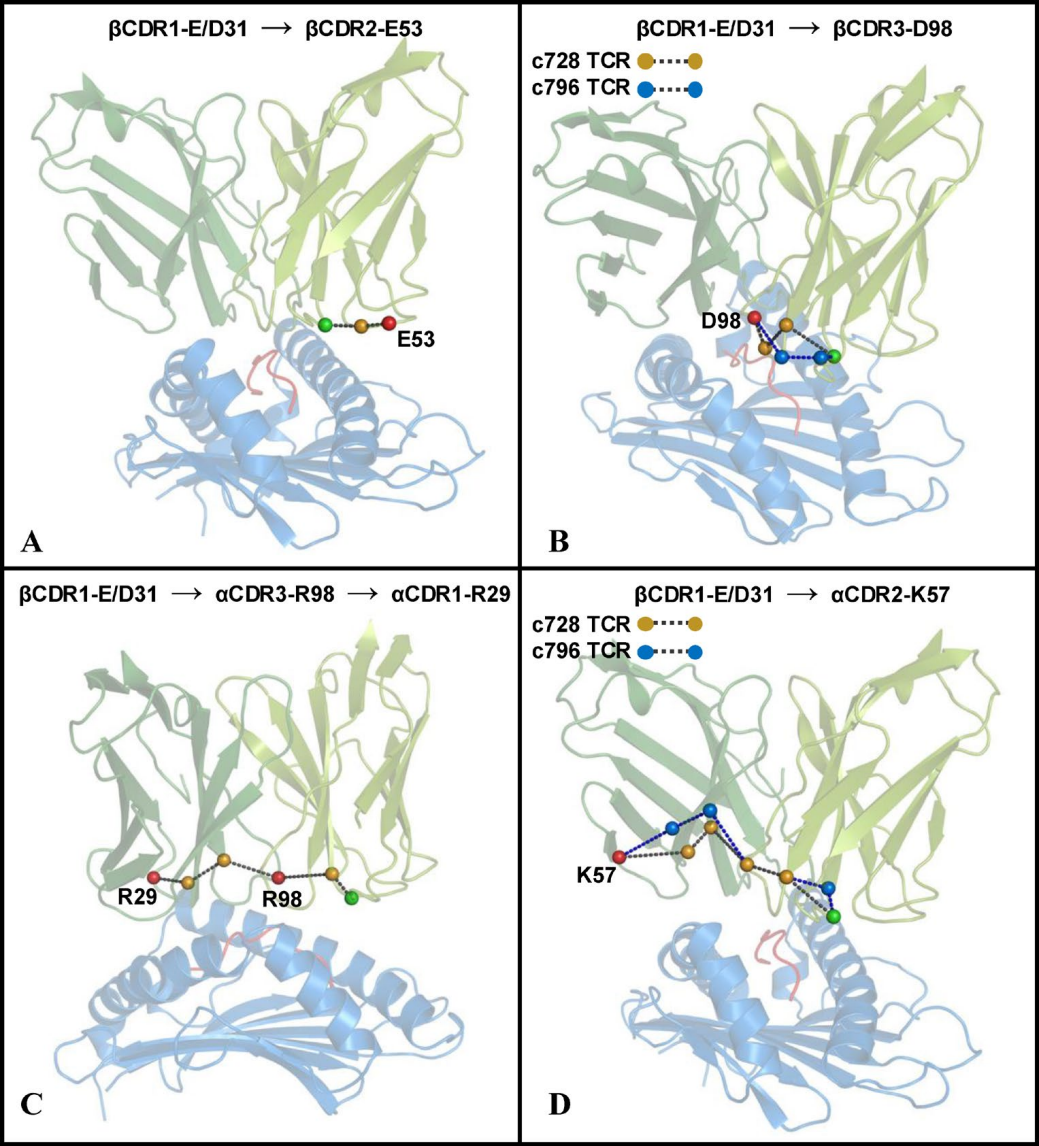
Allosteric pathways. (A) Preferential allosteric pathway from β CDR1‐E31/D31 to β CDR2‐E53. (B) Preferential allosteric pathway from β CDR1‐E31/D31 to β CDR3‐D98. (C) Preferential allosteric pathway from β CDR1‐E31/D31 to α CDR3‐R98 and α CDR1‐R29. (D) Preferential allosteric pathway from β CDR1‐E31/D31 to α CDR2‐K57. The α‐carbon of β CDR1‐E31/D31 is shown as a green sphere, while the α‐carbon of the residue(s) at the endpoint of the allosteric pathway(s) is depicted as a red sphere. The α‐carbon of other residues involved in the allosteric pathway is shown as an orange sphere in both simulated systems if the same preferential pathway was observed. Alternatively, it is shown as an orange sphere for the c728 TCR and a blue sphere for the c796 TCR if distinct preferential allosteric pathways were identified in the two systems.

In this regard, we computed the shortest putative allosteric pathways between β CDR1‐E31/D31 and the aforementioned residues, following the protocol proposed by Ricci et al. [43] (see also the Materials and methods section). These pathways, depicted in Figure 7, were determined using weights corresponding to the inverse of the correlation strength (see Materials and methods section for details). Consequently, lower weights indicate stronger correlations between residues. The total weights of the preferential allosteric pathways originating from β CDR1‐E31/D31, along with the individual weights representing correlations between neighboring residues, are summarized in Table S4.

Our results revealed the presence of a shared pathway in both simulated systems, connecting residue β CDR1‐E31/D31 to residue β CDR2‐E53, characterized by a low global weight (<0.2). In contrast, two distinct pathways were identified for the connection between β CDR1‐E31/D31 and β CDR3‐D98. In the parental TCR, the global weight of this pathway was approximately 0.23, while in the engineered TCR, the weight was slightly higher (≈0.27).

Interestingly, the shortest pathway linking β CDR1‐E31/D31 to α CDR1‐R29 is identical in both TCRs and passes through α CDR3‐R98. Notably, the total weight of the pathway between β CDR1‐E31/D31 and α CDR3‐R98 was found to be significantly low in both systems (≈0.19 and ≈0.24), despite these residues being located on two different polypeptide chains. Finally, the pathway connecting β CDR1‐E31/D31 to α CDR2‐K57, the longest among the residues analyzed, showed slight differences between the two TCRs and exhibited the highest global weights among those computed in this study.

## 3 Discussion

Immunotherapy based on engineered T‐cell receptors represents a promising avenue in cancer treatment, particularly for targeting tumor‐associated antigens [3, 44]. Recent advancements in artificial intelligence [45, 46, 47] have opened new opportunities for identifying TCR modifications that enhance their binding affinity and specificity. However, such approaches, often relying solely on static structural data, fail to account for the dynamic nature of TCR‐pMHC interactions [48]. Integrating molecular dynamics simulations into the design pipeline offers a complementary perspective, providing insights into the flexibility and allosteric mechanisms that govern antigen recognition [31, 36].

Our study presents a test case aimed at rationalizing the enhanced binding affinity observed in an engineered TCR (c796) derived from its parental TCR (c728) combining MD simulations, residue‐specific energy decomposition, and correlation analysis. Importantly, the study is not intended to extrapolate general principles of TCR–pMHC binding, nor can it provide broadly applicable insights into TCR recognition mechanisms. Rather, its goal is to demonstrate how a combination of established computational tools can be applied to a specific engineered system to extract mechanistic and potentially predictive information. The choice of the system was not solely motivated by the fact that the engineered TCR differs from the native one by a single mutation, maintaining an almost unchanged global structure while achieving a six‐fold increase in binding affinity [28]. It was also driven by the system's potential to serve as a study case for improving and evaluating design methods, as well as highlighting the limitations of backbone design approaches, particularly since both ProteinMPNN and ESM‐IF1 failed to generate TCRs with an affinity comparable to that of the native c728 TCR [48].

The structural stability and flexibility of both the parental (c728) and engineered (c796) TCRs, as well as their respective pMHC complexes, were thoroughly evaluated. The RMSD and radius of gyration analyses shown in Figure S1 indicate that both TCR/pMHC complexes maintain overall structural stability throughout the simulated trajectories. Nonetheless, our simulations show that the TCR:pMHC complexes studied in this work require approximately 200ns to reach equilibrium, consistent with the findings of Bingöl et al. [30]. While it is well known that a high number of simulations increases the reliability of results in the study of TCR‐pMHC interactions [18], our results suggest that, depending on the cases studied, such simulations should still be long enough to disregard at least the initial 100–200 ns.

Our work reveals a differential flexibility between the parental and engineered systems. Specifically, the c796 TCR in isolation exhibits reduced flexibility in key regions compared to its parental counterpart, particularly in specific CDR loops involved in antigen binding (see Figure 2B). This reduced flexibility may result in a more stable and energetically favorable binding interface with the pMHC complex. The entropic contribution to binding affinity can be inferred from these flexibility differences. Higher flexibility in binding regions increases entropic penalties upon complex formation [38]. Therefore, the decreased flexibility of the engineered c796 TCR likely contributes to a more favorable entropy change upon binding, complementing the observed energetic advantages. Moreover, the comparison of RMSF values between the TCRs in isolation and in complex with the pMHC provides further insights into the role of conformational rigidity. The engineered TCR displays less pronounced changes in flexibility upon binding (see Figure S4), suggesting a pre‐configured conformation that reduces the entropic cost of forming the TCR/pMHC complex [49]. These observations suggest that optimizing flexibility in engineered TCRs could be a reasonable strategy for balancing enthalpic and entropic contributions to binding affinity, thereby enhancing overall efficacy.

After completing the overall structural analysis, we compared binding energy values derived from static crystallographic structures and molecular dynamics simulations. Our results reveal that, based on x‐ray structures, the parental c728 TCR shows higher affinity for the pMHC complex than the engineered c796 TCR when only short‐range Coulomb and Lennard‐Jones interactions are considered. Conversely, when all Coulomb and Lennard‐Jones interactions are included, the engineered TCR exhibits higher affinity than its parental counterpart (see Table 2).

MD‐based binding energy calculations further reinforce these findings by revealing an even greater affinity for the engineered c796 TCR compared to the parental c728 TCR. By averaging interaction energies over the MD trajectories, we observed enhanced stability in the c796 TCR/pMHC complex, primarily driven by more favorable Coulomb and Lennard‐Jones contributions (see Table 3). These effects are likely a result of conformational adjustments and transient interactions captured only in the dynamic simulations, which are absent in the static crystallographic structures. Furthermore, the solvation energies computed from the MD simulations provided additional insights into the role of dynamic solvent effects in modulating binding affinity. The polar solvation energy is about 110kJ/mol higher in the engineered TCR system when compared to the parental TCR system, suggesting that the solvent effects work against the binding affinity in the engineered system here investigated. This increase in polar solvation energy is a penalty that reduces the overall affinity of the engineered TCR for the pMHC compared to the parental TCR. However, this effect is outweighed by the favorable Coulomb and Lennard‐Jones interactions in the engineered TCR system, which stabilize the complex and contribute more significantly to the increased binding affinity. Although we also performed entropy estimates using the Schlitter approximation, which yielded comparable entropic contributions for the binding of both the engineered and parental TCRs to the pMHC, we chose to focus on relative enthalpic contributions. This decision was motivated by the limitations discussed above: entropic estimates based on quasi‐harmonic approaches are known to suffer from convergence issues and substantial uncertainty in large, flexible systems such as TCR–pMHC complexes. Enthalpic contributions, by contrast, are more robust and interpretable in a comparative context such as the one explored here.

While the qualitative outcome of our results is in line with experimental data, a binding energy difference of 288kJ/mol between the two TCRs and the pMHC is quantitatively excessively high. It is important to note, however, that the MM/PBSA method, despite its widespread use for estimating binding free energies, relies on approximations such as implicit solvation models and limited conformational sampling, which may affect its quantitative accuracy, particularly in flexible protein–protein interfaces like TCR–pMHC complexes. Repeating the calculation using a larger number of independent but shorter replicas, each still sufficiently long to allow discarding the initial 200 ns, reduced this gap to a more reasonable 39.5kJ/mol, further reinforcing the concept that a high number of simulations increases the reliability of results in the study of TCR–pMHC interactions [18].

Our results underscore the importance of integrating a large number of dynamic simulations into the computational pipeline for TCR engineering. While static structural approaches such as docking techniques provide valuable initial insights, they fail to capture the dynamic nature of TCR‐pMHC interactions and can lead to incomplete or inaccurate predictions of binding affinity [50, 51]. Moreover, based on the results obtained from both FEP and MM/PBSA calculations on eight replicas, it is our opinion that the FEP approach, which intrinsically accounts for entropic contributions, should be preferred only when an even higher computational cost than that already employed in the present study can be afforded. In contrast, an MM/PBSA strategy involving multiple replicas of 300–500 ns each may represent a reasonable compromise between computational cost and accuracy when characterizing the binding energy differences of engineered TCRs compared to their parental counterparts.

A key aspect of our study lies in the application of residue‐specific energy decomposition and allosteric pathway analysis to better understand the energetic and dynamic factors that contribute to the enhanced binding affinity of engineered TCRs. By calculating the interaction energies between TCR residues and the pMHC complex, we identified specific residues in the engineered c796 TCR that contribute more significantly to the binding energy compared to the parental c728 TCR. As expected and consistent with previous studies [35], the residues that form stronger interactions with the pMHC complex, thus stabilizing the TCR‐pMHC interface, are located on the CDR loops. However, it is not only the direct interactions between individual residues and the pMHC complex that drive affinity enhancement. Our analysis of the allosteric pathways revealed how the mutation from β CDR1‐E31 to β CDR1‐D31 triggers a network of long‐range allosteric effects that influence the binding characteristics of residues distal to the mutation site. This dynamic interplay between proximal and distal residues significantly contributes to the affinity improvement observed in the engineered TCR. We found that the six‐fold increase in affinity is not directly attributable to the single β CDR1‐E31 to β CDR1‐D31 mutation. Instead, our results suggest that both proximal and distal residues contribute to the observed affinity enhancement through a network of allosteric interactions (see Figures 4 and 7). Specifically, residues within the β CDR2, β CDR3, and α CDR2 loops clearly displayed stronger interactions with the pMHC in the engineered TCR, highlighting the importance of distal effects mediated by conformational changes and allosteric pathways. Notably, residues such as α CDR1‐R29 and α CDR2‐K57 are 20–25 Å away from the mutation site. Nevertheless, our results show that even a single conservative mutation can exert effects at such distances through allosteric mechanisms, consistent with previous observations in other protein systems [52]. Moreover, in some cases, the mutation alters the allosteric pathway between the mutated residue and key sites in the TCR, suggesting a shift in the overall conformational dynamics of the receptor. This change in the allosteric network implies that specific mutations can modify intramolecular signal transmission within the TCR, ultimately influencing its stability and function, as observed in several enzymes [53].

While previous studies have identified global allosteric networks in TCRs, often aiming to explain signal propagation from the extracellular to the intracellular domains [11, 30, 54], our analysis focuses on localized allosteric pathways between functionally relevant sites in two closely related TCR variants. Rather than proposing a universal model of TCR activation, we show how molecular dynamics and correlation‐based network analysis can reveal variant‐specific communication routes that may underlie differences in pMHC binding affinity. These approaches, already applied to TCRs in other contexts, may thus offer a useful framework for the rational design of engineered TCRs. Modulating allosteric pathways can allow for fine‐tuning of TCR specificity and stability, providing a powerful tool for optimizing TCRs for therapeutic applications.

Moreover, this approach may help address the challenge of off‐target toxicity in TCR‐based immunotherapies. Our approach offers a starting point to analyze residue‐specific energetic contributions not only for on‐target pMHCs, but also for potential cross‐reactive peptides presented on healthy cells, potentially minimizing the off‐target toxicity risks. Computational evaluation of such interactions, guided by available peptide‐HLA multimer data or structural modeling, could help identify mutational strategies that weaken unwanted off‐target binding without compromising on‐target affinity. This may be especially relevant in cases where cross‐reactivity arises from structural mimicry at the interface, involving both contact and distal allosteric residues. In this context, energetic decomposition and network analysis, as applied here, may inform fine‐tuning strategies aimed at maximizing specificity while preserving functional potency.

In addition to off‐target recognition, another critical barrier to effective TCR‐based immunotherapy is the immunosuppressive tumor microenvironment (TME). The TME can influence peptide processing and MHC presentation, modulate T cell activation thresholds, and impair TCR signaling via soluble factors, metabolic constraints, or checkpoint inhibition. Although such effects are not directly captured in the present simulation framework, future extensions could integrate models of TCR sensitivity under altered pMHC density, peptide variants, or in the presence of TME‐derived modulators. Moreover, the acidic pH often found in the TME may affect the protonation state of titratable residues at the TCR–pMHC interface, potentially altering binding energetics and specificity. Rational TCR engineering could exploit this property, for example by tuning the ${\mathrm{pK}}_{a}$ of key residues so as to promote productive binding only under tumor‐like pH conditions, thereby reducing affinity or off‐target activation in normal tissues. Although promising, further studies are needed to fully assess the potential for minimizing off‐target toxicity in clinical applications.

While this study focuses on a single engineered TCR–pMHC complex as a test case, yet the computational protocol we applied is inherently general and can be extended to a broader range of systems. Several experimental works have reported SPR‐based binding affinities, including measurement of binding ${\mathrm{\Delta G}}^{{}^{\circ}}$, ${\mathrm{\Delta H}}^{{}^{\circ}}$, and ${\mathrm{\Delta S}}^{{}^{\circ}}$ by van't Hoff analysis, for both wild‐type and engineered TCR variants. For instance, affinity‐enhanced TCRs such as 1G4 targeting NY‐ESO‐1/HLA‐A2 [55] and DMF5 targeting Melan‐A/MART‐1/HLA‐A2 [56] have been reported, along with corresponding SPR‐derived thermodynamic parameters. These systems, whose crystallographic structures have already been resolved, offer excellent opportunities for applying our computational pipeline to multiple targets. In addition, reviews such as the one by Hebeisen et al. [57] summarize a wide range of TCR–pMHC pairs with reported kinetic parameters and mutational data. Similarly, thermodynamic analyses have documented enthalpy–entropy compensation across diverse TCR–pMHC complexes [58], which are particularly suitable to be addressed mechanistically through computational techniques. Leveraging these datasets in tandem with our computational approach could yield deeper insights into the energetic determinants of specificity and affinity in engineered TCRs, thereby aiding structure‐based design for therapeutic optimization. Finally, the residues identified in this study as key contributors to differential binding energy, such as βH30,βE53,andαR98, could serve as starting points for further investigations using computational and experimental alanine scanning mutagenesis. While a full computational alanine scanning campaign would require the repetition of extensive MD and FEP calculations for each point mutation (which is beyond the current study's scope), such simulations could be performed selectively in future work, guided by the residue‐level energetic decomposition we report here. Importantly, this strategy could be directly compared with existing TCR loop mutagenesis data, or used to prioritize minimal experimental validation efforts focused on allosterically connected residues. The combination of targeted mutagenesis and energetic modeling may thus offer a powerful route toward understanding and optimizing engineered TCR–pMHC interfaces.

In perspective, our approach could be integrated into ML‐based pipelines for high‐throughput TCR engineering. Structure prediction and design tools–such as ProteinMPNN and ESM‐IF1, recently tested in the context of TCR design [48], together with frameworks like UniPMT [59], enable rapid screening of mutant libraries, while MD‐based analyses (MM/PBSA, FEP, residue‐level decomposition, correlation networks) provide detailed mechanistic evaluation on prioritized candidates. Beyond affinity enhancement, this strategy may also help reduce cross‐reactivity by identifying mutations that selectively weaken interactions with healthy pMHCs, either directly at the interface or indirectly through distal allosteric effects, without compromising binding to the tumor antigen. Such combined workflows could support the rational design of safer, more specific TCRs.

## 4 Materials and Methods

### 4.1 Molecular Dynamics Simulations

The initial structure of the two complexes formed by human leucocyte antigen (HLA)‐A*02:01 presenting the MAGE‐A10 9‐mer peptide with c728 TCR (PDB ID: 7PDW, 1.82Å resolution) and c796 TCR (PDB ID: 7PBC, 2.04Å resolution) were taken from the protein data bank [29]. Protonation states of all titratable residues were assigned based on empirical ${\mathrm{pK}}_{a}$ prediction carried out with PropKa program [60]. The proton position on the neutral histidine residues was chosen by visual inspection of the structure. The systems were solvated using TIP3p water model [61] in a dodecahedron box imposing a minimum distance between the solute and the box of 1.4nm. Protein residues have been described by the AMBER03 force field [62]. MD simulations were carried out using the GROMACS software package [63, 64]. Long range electrostatic interactions were computed by the Particle Mesh Ewald (PME) method [65], using a grid spacing of 0.12nm and a short range cutoff of 1.2nm. The LINCS algorithm [66] was applied to constrain the bond lengths of the hydrogen atoms to a constant value. A time step of 2fs was used for numerical integration of the equations of motion. The temperature was kept constant at 310K using the V‐rescale algorithm with a coupling time constant $\tau_{T}=0.1\;\mathrm{ps}$ [67] and the system was isotropically coupled to a pressure bath at 1 bar with $\tau_{P}=1.0\;\mathrm{ps}$, using Parrinello‐Rahman barostat [68, 69]. The unrestrained MD simulations were preceded by 50ns of MD simulation with harmonic position restraints (force constant $1000\;\mathrm{kJ}\;{\mathrm{mol}}^{-1}\;{\mathrm{nm}}^{-2}$) on the heavy atoms of protein residues and water oxygen atoms present in the x‐ray structure. For the MD simulations with harmonic position restraints the system was coupled to a pressure bath at 1bar with $\tau_{P}=1.0\;\mathrm{ps}$, using the Berendsen barostat [70]. We simulated three independent trajectories of unrestrained MD simulations (1000ns per trajectory) for all simulated systems starting from the last snapshot of the respective position restrained simulation using different starting velocities. Additionally, ten further independent unrestrained MD trajectories of 500ns each were simulated, five for the parental TCR/pMHC complex and five for the engineered TCR/pMHC complex. The MD simulations of (HLA)‐A*02:01 presenting the MAGE‐A10 9‐mer peptide, TCR c728, and TCR c796 simulated alone, were carried out using the same setup of the two TCR/MHC complexes.

### 4.2 Energy Calculations

The differences in the contributions to the binding energy for the two systems were evaluated using the MM/PBSA method [71, 72, 73]. The MM/PBSA calculations were performed for the whole complex as well as for the TCR and pMHC domains separately, according to the formula:

$$\mathrm{\Delta E}=E_{\mathrm{TOT}}-\left(E_{\mathrm{TCR}}+E_{\text{pMHC}}\right)$$

thus neglecting the bond terms. The energy is divided into three components: internal energy ($\Delta E_{\text{Coul}/\mathrm{LJ}}$), polar solvation energy ($\Delta E_{\text{solv}-\text{polar}}$), and apolar solvation energy ($\Delta E_{\text{solv}-\text{apolar}}$). The internal energy is the sum of all Coulomb (short‐range and reciprocal) and Lennard‐Jones (short‐range and dispersion correction) interactions. The internal energy calculated using only the Lennard‐Jones and Coulomb short‐range interactions is reported as $\Delta E_{\mathrm{SR}}$. The polar solvation contribution was obtained by solving the non‐linear Poisson‐Boltzmann equation using the APBS tool v.1.4.1 [74], with a grid spacing of 0.325Å, a solute dielectric constant of 4, and a solvent dielectric constant of 78.5. The apolar solvation contribution was derived from the Solvent Accessible Surface Area (SASA) using the formula:

$$\Delta E_{\text{apolar}}=\gamma \mathrm{SAS}+b$$

where $\gamma =0.00542^{*}4.184^{*}100\;\mathrm{kJ}/{\mathrm{nm}}^{2}$, $b=0.92^{*}4.184\;\mathrm{kJ}/\mathrm{mol}$ and SAS is the solvent accessible area in ${\mathrm{nm}}^{2}$ [75]. Energy calculations were performed for both the simulated TCR/pMHC complexes, using the crystallographic structures after energy minimization (values reported in Table 2) and for each replica of the MD trajectories (average values reported in Table 3). For the MD trajectories, 800 snapshots were used for the evaluation of the internal and apolar terms, and 80 snapshots for the polar term, excluding the first 200ns of each trajectory. The results for each replica were averaged over the number of snapshots, and finally, the mean of the three replicas was calculated to obtain a single value for each energy contribution.

### 4.3 Free Energy Perturbation Calculations

The binding energy variation upon the E31D mutation between the TCR and the pMHC complex was estimated using free energy perturbation (FEP) calculations following the protocol suggested by the de Groot group [42]. Calculations were performed using the GROMACS software package [63, 64].

Starting from the structural models described in Section 4.1 for the two isolated TCRs and the two TCR/pMHC complexes, hybrid topologies were generated for all four systems using the pmx tool [76]. After energy minimization, each system underwent a position‐restrained MD simulation applied to heavy atoms. Subsequently, three independent unrestrained MD simulations of 210 ns each were performed in the NPT ensemble for each system. From the last 10 ns of each trajectory, 100 frames were extracted at 100 ps intervals, yielding 300 starting configurations per system. From these configurations, we carried out 300 nonequilibrium FEP simulations per system in the forward direction (mutation from glutamic acid to aspartic acid, corresponding to λ variation from 0 to 1) and 300 in the backward direction (mutation from aspartic acid to glutamic acid, λ variation from 1 to 0), resulting in a total of 1200 nonequilibrium FEP simulations across the four systems. In each of these nonequilibrium simulations, the λ parameter was gradually changed over 200 000 integration steps with a time step of 0.5 fs. Soft‐core potentials were employed during the alchemical transformation, using a soft‐core alpha value of 0.3 and a soft‐core sigma value of 0.25 nm. The results were analyzed with the tool *pmx analyze*, and the resulting $\Delta \Delta G_{b}^{\mathrm{eng}-\mathrm{par}}$ was calculated as reported in Figure 3.

### 4.4 TCR‐MHC Pairwise Residue Energy Calculations

Residue‐to‐residue energy calculations (Coulomb and Lennard‐Jones short‐range interactions) were performed between the residues of the TCR domain and those of the MAGE‐A10 peptide, as well as the two α helices in the binding groove subdomain of the MHC. These calculations were based on 800 frames extracted from each trajectory, excluding the first 200ns of equilibration. Results were averaged across the three replicas for each system. To identify significant interactions, a contact map was defined. Specifically, a residue in the TCR domain was considered to be in contact with a residue in the two α helices of the binding groove and/or the MAGE‐A10 peptide if any pair of their respective atoms was within a distance of 6Å in at least one frame. For residue pairs identified by the contact map, short‐range energy calculations were carried out by rerunning the MD simulations and instructing the GROMACS tool to compute the energy contributions specifically for the relevant residue pairs.

### 4.5 Correlation Analysis

The inter‐residue correlation analysis was performed using the non‐linear Generalized Correlation method [77], as implemented in the g_correlation tool on top of GROMACS v. 3.3 [63]. The computation was conducted on the $C_{\alpha }$ atoms, excluding the first 200ns of each production run. The resulting correlation matrices were used to construct inter‐residue networks with an in‐house Python script leveraging the NetworkX library [78]. Shortest, putative allosteric paths were computed using the Dijkstra algorithm, as implemented in NetworkX, with weights defined as the inverse of the correlation strength w, expressed as $-\log_{10}\left(w\right)$. To reduce noise, the correlation matrices were filtered using a contact map, which defined residues as being in contact if any pair of their heavy atoms was within 5Å for at least 70% of the frames [79]. The contact map was generated using an in‐house Python script employing the MDAnalysis library [80, 81] for distance calculations. All correlation results were obtained separately for each replica from the trajectories of the c728 TCR–HLA‐A*02:01/MAGE‐A10 and c796 TCR–HLA‐A*02:01/MAGE‐A10 complexes. The results were then averaged across the three replicas.

### 4.6 Trajectory Analysis

Graphical and numerical analyses of the trajectories were performed using VMD v. 11.9.3 [82] and tools from the GROMACS suite [83]. Molecular structures were visualized and rendered with the open‐source version of PyMOL v. 12.5 [84]. Custom Python and Bash scripts were developed as needed for specific analyses. All dynamical plots presented in the figures were generated using Gnuplot v. 15.2 [85]. All residue indices reported in the analysis, figures, and throughout the text refer to the residue numbering found in the original PDB structures (PDB IDs: 7PDW and 7PBC).

## Author Contributions

**Mario Frezzini:** software, validation, visualization, writing – original draft, writing – review and editing, investigation, formal analysis, methodology, data curation. **Daniele Narzi:** conceptualization, investigation, resources, writing – review and editing, writing – original draft, supervision, data curation.

## Conflicts of Interest

The authors declare no conflicts of interest.

## Supporting information


**Data S1:** Supporting Information.

## Data Availability

All MD simulations were produced with GROMACS (https://www.gromacs.org). Generalized correlations were computed with the tool g_correlation for the GROMACS framework (https://www.mpinat.mpg.de/grubmueller/g_correlation). Trajectories were analyzed with VMD (https://www.ks.uiuc.edu/Research/vmd) and the Python libraries MDAnalysis (https://www.mdanalysis.org) and NetworkX (https://networkx.org). GROMACS is available under the GNU Lesser General Public License (LGPL), version 2.1 (https://www.gnu.org/licenses/old‐licenses/lgpl‐2.1.html). VMD is distributed free of charge for nonexclusive, noncommercial use (https://www.ks.uiuc.edu/Research/vmd/current/LICENSE.html). MDAnalysis is available under the GNU General Public License (GPL), version 2.0 (https://www.gnu.org/licenses/old‐licenses/gpl‐2.0.html). NetworkX is distributed with the 3‐clause BSD license (https://opensource.org/license/BSD‐3‐clause). Coordinates (pdb), topology (top, itp), index (ndx) files of each system described in this work, and molecular dynamics parameters (mdp) files used for free MD simulations are publicly available on Zenodo at: https://zenodo.org/records/14811023. Each additional file can be provided by the authors upon reasonable request.

## References

[1] P. A. van der Merwe and S. J. Davis , “Molecular Interactions Mediating T Cell Antigen Recognition,” Annual Review of Immunology 21 (2003): 659–684, 10.1146/annurev.immunol.21.120601.141036. 12615890

[2] D. N. Garboczi , P. Ghosh , U. Utz , Q. R. Fan , W. E. Biddison , and D. C. Wiley , “Structure of the Complex Between Human T‐Cell Receptor, Viral Peptide and HLA‐A2,” Nature 384 (1996): 134–141, 10.1038/384134a0. 8906788

[3] A. D. Waldman , J. M. Fritz , and M. J. Lenardo , “A Guide to Cancer Immunotherapy: From T Cell Basic Science to Clinical Practice,” Nature Reviews. Immunology 20 (2020): 651–668, 10.1038/s41577-020-0306-5. PMC7238960 32433532

[4] P. N. Kelly , “The Cancer Immunotherapy Revolution,” Science 359 (2018): 1344–1345, 10.1126/science.359.6382.134. 29567702

[5] D. Li , X. Li , W. L. Zhou , et al., “Genetically Engineered T Cells for Cancer Immunotherapy,” Signal Transduction and Targeted Therapy 4 (2019): 35, 10.1038/s41392-019-0070-9. 31637014 PMC6799837

[6] K. C. Garcia , M. Degano , L. R. Pease , et al., “Structural Basis of Plasticity in T Cell Receptor Recognition of a Self Peptide‐MHC Antigen,” Science 279, no. 5354 (1998): 1166–1172, 10.1126/science.279.5354.1166. 9469799

[7] F. E. Tynan , H. H. Reid , L. Kjer‐Nielsen , et al., “A T Cell Receptor Flattens a Bulged Antigenic Peptide Presented by a Major Histocompatibility Complex Class I Molecule,” Nature Immunology 8 (2007): 268–276, 10.1038/ni1432. 17259989

[8] D. K. Sethi , S. Gordo , D. A. Schubert , and K. W. Wucherpfennig , “Crossreactivity of a Human Autoimmune TCR Is Dominated by a Single TCR Loop,” Nature Communications 4 (2013): 2623, 10.1038/ncomms3623. PMC4193804 24136005

[9] P. Zareie , C. Szeto , C. Farenc , et al., “Canonical T Cell Receptor Docking on Peptide–MHC Is Essential for T Cell Signaling,” Science 372 (2021): eabe9124, 10.1126/science.abe9124. 34083463

[10] W. Hawse , M. Champion , M. Joyce , et al., “Cutting Edge: Evidence for a Dynamically Driven T Cell Signaling Mechanism,” Journal of Immunology 188 (2012): 5819–5823, 10.4049/jimmunol.1200952. PMC3375328 22611242

[11] S. Rangarajan , Y. He , Y. Chen , et al., “Peptide‐MHC (pMHC) Binding to a Human Antiviral T Cell Receptor Induces Long‐Range Allosteric Communication Between pMHC‐ and CD3‐Binding Sites,” Journal of Biological Chemistry 293 (2018): 15991–16005, 10.1074/jbc.RA118.003832. 30135211 PMC6187629

[12] K. Natarajan , J. Jiang , N. A. May , et al., “The Role of Molecular Flexibility in Antigen Presentation and T Cell Receptor‐Mediated Signaling,” Frontiers in Immunology 9 (2018): 1657, 10.3389/fimmu.2018.01657. 30065727 PMC6056622

[13] J. Alba , L. D. Rienzo , E. Milanetti , O. Acuto , and M. D'Abramo , “Molecular Dynamics Simulations Reveal Canonical Conformations in Different pMHC/TCR Interactions,” Cells 10 (2020): 942, 10.3390/cells9040942. PMC7226950 32290289

[14] J. Alba and M. D'Abramo , “The Full Model of the pMHC‐TCR‐CD3 Complex: A Structural and Dynamical Characterization of Bound and Unbound States,” Cells 11, no. 4 (2022): 668, 10.3390/cells11040668. 35203317 PMC8869815

[15] C. F. Reboul , G. R. Meyer , B. T. Porebski , N. A. Borg , and A. M. Buckle , “Epitope Flexibility and Dynamic Footprint Revealed by Molecular Dynamics of a pMHC‐TCR Complex,” PLoS Computational Biology 8, no. 3 (2012): 1–11, 10.1371/journal.pcbi.1002404. 22412359 PMC3297556

[16] B. Knapp , J. Dunbar , and C. M. Deane , “Large Scale Characterization of the LC13 TCR and HLA‐B8 Structural Landscape in Reaction to 172 Altered Peptide Ligands: A Molecular Dynamics Simulation Study,” PLoS Computational Biology 10, no. 8 (2014): 1–14, 10.1371/journal.pcbi.1003748. PMC4125040 25101830

[17] H. Zhang , H. S. Lim , B. Knapp , et al., “The Contribution of Major Histocompatibility Complex Contacts to the Affinity and Kinetics of T Cell Receptor Binding,” Scientific Reports 6, no. 1 (2016): 35326, 10.1038/srep35326. 27734930 PMC5062128

[18] J. L. Dominguez and B. Knapp , “How Peptide/MHC Presence Affects the Dynamics of the LC13 T‐Cell Receptor,” Scientific Reports 9, no. 1 (2019): 2638, 10.1038/s41598-019-38788-0. 30804417 PMC6389892

[19] B. Knapp , v. d P A. Merwe , O. Dushek , and C. M. Deane , “MHC Binding Affects the Dynamics of Different T‐Cell Receptors in Different Ways,” PLoS Computational Biology 15, no. 9 (2019): 1–17, 10.1371/journal.pcbi.1007338. PMC6752857 31498801

[20] R. Karch , C. Stocsits , N. Ilieva , and W. Schreiner , “Intramolecular Domain Movements of Free and Bound pMHC and TCR Proteins: A Molecular Dynamics Simulation Study,” Cells 8, no. 7 (2019): 720, 10.3390/cells8070720. 31337065 PMC6678086

[21] L. Tomasiak , R. Karch , and W. Schreiner , “Conformational Flexibility of a Free and TCR‐Bound pMHC‐I Protein Investigated by Long‐Term Molecular Dynamics Simulations,” BMC Immunology 23, no. 1 (2022): 36, 10.1186/s12865-022-00510-7. 35902791 PMC9335952

[22] M. Ferber , V. Zoete , and O. Michielin , “T‐Cell Receptors Binding Orientation Over Peptide/MHC Class I Is Driven by Long‐Range Interactions,” PLoS One 7, no. 12 (2012): 1–15, 10.1371/journal.pone.0051943. PMC3522592 23251658

[23] R. C. Eccleston , S. Wan , N. Dalchau , and P. V. Coveney , “The Role of Multiscale Protein Dynamics in Antigen Presentation and T Lymphocyte Recognition,” Frontiers in Immunology 8 (2017): 8, 10.3389/fimmu.2017.00797. 28740497 PMC5502259

[24] Z. A. Rollins , R. Faller , and S. C. George , “Using Molecular Dynamics Simulations to Interrogate T Cell Receptor Non‐Equilibrium Kinetics,” Computational and Structural Biotechnology Journal 20 (2022): 2124–2133, 10.1016/j.csbj.2022.04.018. 35832631 PMC9092387

[25] T. Lu , Z. Zhang , J. Zhu , et al., “Deep Learning‐Based Prediction of the T Cell Receptor–Antigen Binding Specificity,” Nature Machine Intelligence 3 (2021): 864–875, 10.1038/s42256-021-00383-2. PMC9396750 36003885

[26] J. Bujak , S. Kłek , M. Balawejder , et al., “Creating an Innovative Artificial Intelligence‐Based Technology (TCRact) for Designing and Optimizing T Cell Receptors for Use in Cancer Immunotherapies: Protocol for an Observational Trial,” JMIR Research Protocols 12 (2023): e45872, 10.2196/45872. 37440307 PMC10375398

[27] P. Asadi Sarabi , M. Shabanpouremam , A. R. Eghtedari , et al., “AI‐Based Solutions for Current Challenges in Regenerative Medicine,” European Journal of Pharmacology 984 (2024): 177067, 10.1016/j.ejphar.2024.177067. 39454850

[28] E. C. Border , J. P. Sanderson , T. Weissensteiner , A. B. Gerryb , and N. J. Pumphrey , “Affinity‐Enhanced T‐Cell Receptors for Adoptive T‐Cell Therapy Targeting MAGE‐A10: Strategy for Selection of an Optimal Candidate,” Oncoimmunology 8 (2019): e1532759, 10.1080/2162402X.2018.1532759. 30713784 PMC6343776

[29] P. C. Simister , E. C. Border , J. F. Vieira , and N. J. Pumphrey , “Structural Insights Into Engineering a T‐Cell Receptor Targeting MAGE‐A10 With Higher Affinity and Specificity for Cancer Immunotherapy,” Journal for ImmunoTherapy of Cancer 10 (2022): e004600, 10.1136/jitc-2022-004600. 35851311 PMC9295655

[30] E. N. Bingöl , O. Serçinoğlu , and P. Ozbek , “Unraveling the Allosteric Communication Mechanisms in T‐Cell Receptor–Peptide‐Loaded Major Histocompatibility Complex Dynamics Using Molecular Dynamics Simulations: An Approach Based on Dynamic Cross Correlation Maps and Residue Interaction Energy Calculations,” Journal of Chemical Information and Modeling 61, no. 5 (2021): 2444–2453, 10.1021/acs.jcim.1c00338. 33930270

[31] T. P. Riley , C. M. Ayres , L. M. Hellman , et al., “A Generalized Framework for Computational Design and Mutational Scanning of T‐Cell Receptor Binding Interfaces,” Protein Engineering, Design & Selection 29, no. 12 (2016): 595–606, 10.1093/protein/gzw050. PMC5181382 27624308

[32] P. S. Merkle , M. Irving , S. Hongjian , et al., “The T‐Cell Receptor Can Bind to the Peptide‐Bound Major Histocompatibility Complex and Uncomplexed β2−Microglobulin Through Distinct Binding Sites,” Biochemistry 56, no. 30 (2017): 3945–3961, 10.1021/acs.biochem.7b00385. 28671821

[33] V. Apostolopoulos , G. Deraos , M. T. Matsoukas , et al., “Cyclic Citrullinated MBP87–99 Peptide Stimulates T Cell Responses: Implications in Triggering Disease,” Bioorganic & Medicinal Chemistry 25, no. 2 (2017): 528–538, 10.1016/j.bmc.2016.11.029. 27908754

[34] P. Wu , T. Zhang , B. Liu , et al., “Mechano‐Regulation of Peptide‐MHC Class I Conformations Determines TCR Antigen Recognition,” Molecular Cell 73, no. 5 (2019): 1015–1027.e7, 10.1016/j.molcel.2018.12.018. 30711376 PMC6408234

[35] R. M. Crean , B. J. MacLachlan , F. Madura , et al., “Molecular Rules Underpinning Enhanced Affinity Binding of Human T Cell Receptors Engineered for Immunotherapy,” Molecular Therapy ‐ Oncolytics 18 (2020): 443–456, 10.1016/j.omto.2020.07.008. 32913893 PMC7452143

[36] Z. A. Rollins , M. B. Curtis , S. C. George , and R. Faller , “A Computational Strategy for the Rapid Identification and Ranking of Patient‐Specific T Cell Receptors Bound to Neoantigens,” Macromolecular Rapid Communications 45, no. 24 (2024): 2400225, 10.1002/marc.202400225. 38839076 PMC11661661

[37] J. Ma , C. M. Ayres , C. A. Brambley , et al., “Dynamic Allostery in the Peptide/MHC Complex Enables TCR Neoantigen Selectivity,” Nature Commununications 16 (2025): 849, 10.1038/s41467-025-56004-8. PMC11756396 39833157

[38] D. Narzi , C. M. Becker , M. T. Fiorillo , B. Uchanska‐Ziegler , A. Ziegler , and R. A. Böckmann , “Dynamical Characterization of Two Differentially Disease Associated MHC Class I Proteins in Complex With Viral and Self‐Peptides,” Journal of Molecular Biology 415 (2012): 429–442, 10.1016/j.jmb.2011.11.021. 22119720

[39] J. Schlitter , “Estimation of Absolute and Relative Entropies of Macromolecules Using the Covariance Matrix,” Chemical Physics Letters 215 (1993): 617–621.

[40] Z. Xia , H. Chen , S. Kang , et al., “The Complex and Specific pMHC Interactions With Diverse HIV‐1 TCR Clonotypes Reveal a Structural Basis for Alterations in CTL Function,” Scientific Reports 4 (2014): 4087. 24522437 10.1038/srep04087 PMC3923210

[41] Y. Song , D. R. Bell , R. Ahmed , et al., “A Mutagenesis Study of Autoantigen Optimization for Potential T1D Vaccine Design,” National Academy of Sciences of the United States of America 120 (2023): e2214430120. 10.1073/pnas.2214430120 PMC10120010 37040399

[42] M. Aldeghi , B. L. de Groot , and V. Gapsys , “Accurate Calculation of Free Energy Changes Upon Amino Acid Mutation,” Methods in Molecular Biology 1851 (2018): 19–47, 10.1007/978-1-4939-8736-8_2. 30298390

[43] C. G. Ricci , R. L. Silveira , I. Rivalta , V. S. Batista , and M. S. Skaf , “Allosteric Pathways in the PPARγ−RXRα Nuclear Receptor Complex,” Scientific Reports 6, no. 1 (2016): 19940, 10.1038/srep19940. 26823026 PMC4731802

[44] Q. Zhao , Y. Jiang , S. Xiang , et al., “Engineered TCR‐T Cell Immunotherapy in Anticancer Precision Medicine: Pros and Cons,” Frontiers in Immunology 12 (2021): 658753, 10.3389/fimmu.2021.658753. 33859650 PMC8042275

[45] Z. Lin , H. Akin , R. Rao , et al., “Evolutionary‐Scale Prediction of Atomic‐Level Protein Structure With a Language Model,” Science 379 (2023): 1123–1130, 10.1126/science.ade2574. 36927031

[46] C. Hsu , R. Verkuil , J. Liu , et al., “Learning Inverse Folding From Millions of Predicted Structures,” in 162 of Proceedings of Machine Learning Research. Proceedings of Machine Learning Research (PMLR, 2022), 8946–8970.

[47] J. Dauparas , I. Anishchenko , N. Bennett , et al., “Robust Deep Learning–Based Protein Sequence Design Using ProteinMPNN,” Science 378 (2022): 49–56, 10.1126/science.add2187. 36108050 PMC9997061

[48] H. V. Ribeiro‐Filho , G. E. Jara , J. V. S. Guerra , et al., “Exploring the Potential of Structure‐Based Deep Learning Approaches for T Cell Receptor Design,” PLoS Computational Biology 20 (2024): e1012489, 10.1371/journal.pcbi.1012489. 39348412 PMC11466415

[49] C. J. Holland , P. J. Rizkallah , S. Vollers , et al., “Minimal Conformational Plasticity Enables TCR Cross‐Reactivity to Different MHC Class II Heterodimers,” Scientific Reports 2 (2012): 629, 10.1038/srep00629. 22953050 PMC3432979

[50] F. Ambrosetti , B. Jiménez‐García , J. Roel‐Touris , and A. M. J. J. Bonvin , “Modeling Antibody‐Antigen Complexes by Information‐Driven Docking,” Structure 28 (2020): 119–129, 10.1016/j.str.2019.10.011. 31727476

[51] T. Peacock and B. Chain , “Information‐Driven Docking for TCR‐pMHC Complex Prediction,” Frontiers in Immunology 12 (2021): 686127, 10.3389/fimmu.2021.686127. 34177934 PMC8219952

[52] N. Rajasekaran , A. Sekhar , and A. N. Naganathan , “A Universal Pattern in the Percolation and Dissipation of Protein Structural Perturbations,” Journal of Physical Chemistry Letters 8, no. 19 (2017): 4779–4784, 10.1021/acs.jpclett.7b02021. 28910120

[53] A. N. Naganathan , “Modulation of Allosteric Coupling by Mutations: From Protein Dynamics and Packing to Altered Native Ensembles and Function,” Current Opinion in Structural Biology 54 (2019): 1–9, 10.1016/j.sbi.2018.09.004. 30268910 PMC6420056

[54] Y. He , P. Agnihotri , S. Rangarajan , et al., “Peptide–MHC Binding Reveals Conserved Allosteric Sites in MHC Class I‐ and Class II‐Restricted T Cell Receptors (TCRs),” Journal of Molecular Biology 432 (2020): 166697. 33157083 10.1016/j.jmb.2020.10.031 PMC8356565

[55] Y. Zhao , A. D. Bennett , Z. Zheng , et al., “High‐Affinity TCRs Generated by Phage Display Provide CD4+ T Cells With the Ability to Recognize and Kill Tumor Cell Lines1,” Journal of Immunology (Baltimore, Md.: 1950) 179 (2007): 5845–5854. 17947658 10.4049/jimmunol.179.9.5845 PMC2140228

[56] L. A. Johnson , B. Heemskerk , D. J. J. Powell , et al., “Gene Transfer of Tumor‐Reactive TCR Confers Both High Avidity and Tumor Reactivity to Nonreactive Peripheral Blood Mononuclear Cells and Tumor‐Infiltrating Lymphocytes,” Journal of Immunology (Baltimore, Md.: 1950) 177 (2006): 6548–6559. 17056587 10.4049/jimmunol.177.9.6548 PMC2174608

[57] M. Hebeisen , M. Allard , P. O. Gannon , J. Schmidt , D. E. Speiser , and N. Rufer , “Identifying Individual T Cell Receptors of Optimal Avidity for Tumor Antigens,” Frontiers in Immunology 6 (2015): 582. 26635796 10.3389/fimmu.2015.00582 PMC4649060

[58] K. M. Armstrong , F. K. Insaidoo , and B. M. Baker , “Thermodynamics of T‐Cell Receptor‐Peptide/MHC Interactions: Progress and Opportunities,” Journal of Molecular Recognition 21 (2008): 275–287. 18496839 10.1002/jmr.896 PMC3674762

[59] Y. Zhao , J. Yu , Y. Su , et al., “A Unified Deep Framework for Peptide–Major Histocompatibility Complex–T Cell Receptor Binding Prediction,” Nature Machine Intelligence 7 (2025): 650–660.

[60] H. Li , A. D. Robertson , and J. H. Jensen , “Very Fast Empirical Prediction and Rationalization of Protein pKa Values,” Proteins: Structure, Function, and Bioinformatics 61, no. 4 (2005): 704–721, 10.1002/prot.20660. 16231289

[61] W. L. Jorgensen , J. Chandrasekhar , J. D. Madura , R. W. Impey , and M. L. Klein , “Comparison of Simple Potential Functions for Simulating Liquid Water,” Journal of Chemical Physics 79, no. 2 (1983): 926–935, 10.1063/1.445869.

[62] Y. Duan , C. Wu , S. Chowdhury , et al., “A Point‐Charge Force Field for Molecular Mechanics Simulations of Proteins Based on Condensed‐Phase Quantum Mechanical Calculations,” Journal of Computational Chemistry 24 (2003): 1999–2012, 10.1002/jcc.10349. 14531054

[63] E. Lindahl , B. Hess , and v. d D. Spoel , “GROMACS 3.0: A Package for Molecular Simulation and Trajectory Analysis,” Ournal of Molecular Modeling 7, no. 8 (2001): 306–317, 10.1007/s008940100045.

[64] D. van der Spoel , E. Lindahl , B. Hess , G. Groenhof , A. Mark , and H. Berendsen , “GROMACS: Fast, Flexible, and Free,” Journal of Computational Chemistry 26 (2005): 1701–1718, 10.1002/jcc.20291. 16211538

[65] T. A. Darden , D. M. York , and L. Pedersen , “Particle Mesh Ewald: An $N\log\left(N\right)$ Method for Ewald Sums in Large Systems,” Journal of Chemical Physics 98 (1993): 10089–10092, 10.1063/1.464397.

[66] B. Hess , H. Bekker , H. J. C. Berendsen , and J. G. E. M. Fraaije , “LINCS: A Linear Constraint Solver for Molecular Simulations,” Journal of Computational Chemistry 18, no. 12 (1997): 1463–1472, 10.1002/(SICI)1096-987X(199709)18:12<1463::AID-JCC4>3.0.CO;2-H.

[67] G. Bussi , D. Donadio , and M. Parrinello , “Canonical Sampling Through Velocity Rescaling,” Journal of Chemical Physics 126, no. 1 (2007): 014101, 10.1063/1.2408420. 17212484

[68] M. Parrinello and A. Rahman , “Polymorphic Transitions in Single Crystals: A New Molecular Dynamics Method,” Journal of Applied Physics 52, no. 12 (1981): 7182–7190, 10.1063/1.328693.

[69] S. Nosé and M. L. Klein , “Constant Pressure Molecular Dynamics for Molecular Systems,” Molecular Physics 50 (1983): 1055–1076, 10.1080/00268978300102851.

[70] H. J. C. Berendsen , J. P. M. Postma , v W F. Gunsteren , A. DiNola , and J. R. Haak , “Molecular Dynamics With Coupling to an External Bath,” Journal of Chemical Physics 81, no. 8 (1984): 3684–3690, 10.1063/1.448118.

[71] J. Srinivasan , T. E. Cheatham , P. Cieplak , P. A. Kollman , and D. A. Case , “Continuum Solvent Studies of the Stability of DNA, RNA, and Phosphoramidate‐DNA Helices,” Journal of the American Chemical Society 120, no. 37 (1998): 9401–9409, 10.1021/ja981844+.

[72] P. A. Kollman , I. Massova , C. Reyes , et al., “Calculating Structures and Free Energies of Complex Molecules: Combining Molecular Mechanics and Continuum Models,” Accounts of Chemical Research 33, no. 12 (2000): 889–897, 10.1021/ar000033j. 11123888

[73] S. Genheden and U. Ryde , “The MM/PBSA and MM/GBSA Methods to Estimate Ligand‐Binding Affinities,” Expert Opinion on Drug Discovery 10, no. 5 (2015): 449–461, 10.1517/17460441.2015.1032936. 25835573 PMC4487606

[74] N. A. Baker , D. Sept , S. Joseph , M. J. Holst , and J. A. McCammon , “Electrostatics of Nanosystems: Application to Microtubules and the Ribosome,” Proceedings of the National Academy of Sciences of the United States of America 98, no. 18 (2001): 10037–10041, 10.1073/pnas.181342398. 11517324 PMC56910

[75] I. Massova and P. A. Kollman , “Computational Alanine Scanning to Probe Protein‐Protein Interactions: A Novel Approach to Evaluate Binding Free Energies,” Journal of the American Chemical Society 121, no. 36 (1999): 8133–8143, 10.1021/ja990935j.

[76] V. Gapsys , S. Michielssens , D. Seeliger , and B. L. de Groot , “Pmx: Automated Protein Structure and Topology Generation for Alchemical Perturbations,” Journal of Computational Chemistry 36 (2015): 348–354. 25487359 10.1002/jcc.23804 PMC4365728

[77] O. F. Lange and H. Grubmüller , “Generalized Correlation for Biomolecular Dynamics,” Proteins: Structure, Function, and Bioinformatics 62, no. 4 (2006): 1053–1061, 10.1002/prot.20784. 16355416

[78] A. Hagberg , D. Schult , and P. Swart , “Exploring Network Structure, Dynamics, and Function Using NetworkX,” in 7th Python in Science Conference, ed. G. Varoquaux , T. Vaught , and J. Millman (2008), 11–16.

[79] K. W. East , J. C. Newton , U. N. Morzan , et al., “Allosteric Motions of the CRISPR–Cas9 HNH Nuclease Probed by NMR and Molecular Dynamics,” Journal of the American Chemical Society 142, no. 3 (2020): 1348–1358, 10.1021/jacs.9b10521. 31885264 PMC7497131

[80] N. Michaud‐Agrawal , E. J. Denning , T. B. Woolf , and O. Beckstein , “MDAnalysis: A Toolkit for the Analysis of Molecular Dynamics Simulations,” Journal of Computational Chemistry 32, no. 10 (2011): 2319–2327, 10.1002/jcc.21787. 21500218 PMC3144279

[81] R. J. Gowers , M. Linke , J. Barnoud , et al., “MDAnalysis: A Python Package for the Rapid Analysis of Molecular Dynamics Simulations,” in Sebastian Benthall, Scott Rostrup, editors. Proceedings of the 15th Python in Science Conference (2016), 98–105.

[82] W. Humphrey , A. Dalke , and K. Schulten , “VMD: Visual Molecular Dynamics,” Journal of Molecular Graphics 14, no. 1 (1996): 33–38, 10.1016/0263-7855(96)00018-5. 8744570

[83] H. Berendsen , D. van der Spoel , and R. van Drunen , “GROMACS: A Message‐Passing Parallel Molecular Dynamics Implementation,” Computer Physics Communications 91, no. 1 (1995): 43–56, 10.1016/0010-4655(95)00042-E.

[84] W. L. DeLano , “PyMOL: An Open‐Source Molecular Graphics Tool,” CCP4 Newsletter on Protein Crystallography 40, no. 11 (2002): 82–92.

[85] T. Williams and C. Kelley , “Gnuplot 5.2: An Interactive Plotting Program.” 2019, http://gnuplot.sourceforge.net/.

