# Supplementary material for: Rationalizing Enhanced Affinity of Engineered T‐Cell Receptors in Cancer Immunotherapy Through Interaction Energy Calculations and Residue Correlation Analysis
Source: Proteins. 2025 Aug 1;94(2):529–46. doi: 10.1002/prot.70028 (PMC12779239; doi:10.1002/prot.70028)
Supplement: Supplementary file 1 — Data S1: Supporting Information. [file PROT-94-529-s001.pdf]

Received: Added at production | Revised: Added at production | Accepted: Added at production
DOI: xxx/xxxx

SUPPORTING INFORMATION

Rationalizing Enhanced Affinity of Engineered T-Cell Receptors in Cancer Immunotherapy through Interaction Energy Calculations and Residue Correlation Analysis

Mario Frezzini[1] | **Daniele Narzi**[2]

[1]Department of Information Engineering, Computer Science and Mathematics, University of L'Aquila, L'Aquila, 67100, Italy

[2]Department of Physical and Chemical Sciences, University of L'Aquila, L'Aquila, 67100, Italy

Correspondence
Daniele Narzi, Department of Physical and Chemical Sciences, University of L'Aquila, L'Aquila, 67100, Italy.
Email: daniele.narzi@univaq.it

 © 2025 Copyright Holder Name

TABLE S1 Entropy and −TS contributions calculated for each of the three independent 1000 ns MD replicas. The first column reports the replica index (R1, R2, R3). Columns 2 to 5 report the Schlitter entropy estimates (in kJ/mol·K) for the parental TCR, engineered TCR, parental TCR/pMHC complex, and engineered TCR/pMHC complex, respectively. Columns 6 to 9 report the corresponding −TS values (in kJ/mol) calculated at T=310 K for each system. The Schlitter entropy was computed considering all heavy atoms of the isolated TCRs, whereas for the TCR/pMHC complexes, the calculation included all heavy atoms of the TCR and those of the binding groove of the pMHC together with the presented peptide.

Replica	c728 TCR S (kJ/mol K)	c796 TCR S (kJ/mol K)	c728/pMHC S (kJ/mol K)	c796/pMHC S (kJ/mol K)	c728 TCR -TS (kJ/mol)	c796 TCR -TS (kJ/mol)	c728/pMHC -TS (kJ/mol)	c796/pMHC -TS (kJ/mol)
R1	40.14	40.12	56.44	56.73	-12443.4	-12437.2	-17496.4	-17586.3
R2	40.78	40.05	57.53	57.05	-12641.8	-12415.5	-17834.3	-17685.5
R2	40.55	40.95	57.09	57.11	-12570.5	-12694.5	-17697.9	-17704.1

TABLE S2 TCR-pMHC binding energy averaged over eight 500 ns-long simulated trajectories. Standard deviations of the mean are provided in parentheses. Values are reported in kJ/mol. For further details, refer to Section Energy calculations.

	c728 TCR HLA-A*02:01/MAGE-A10	c796 TCR HLA-A*02:01/MAGE-A10
$\langle \Delta E_{SR} \rangle_{MD}$ ($\langle \sigma_\mu \rangle_{MD}$)	-747.0 (11.4)	-768.1 (27.0)
$\langle \Delta E_{Coul/LJ} \rangle_{MD}$ ($\langle \sigma_\mu \rangle_{MD}$)	-980.9 (25.8)	-1060.7 (42.3)
$\langle \Delta E_{solv-polar} \rangle_{MD}$ ($\langle \sigma_\mu \rangle_{MD}$)	497.4 (14.6)	537.4 (21.3)
$\langle \Delta E_{solv-apolar} \rangle_{MD}$ ($\langle \sigma_\mu \rangle_{MD}$)	-49.0 (0.4)	-48.7 (0.9)

TABLE S3 Statistics of significant inter-residue contacts averaged over three 1000 ns-long simulated trajectories (the first 200 ns have been neglected) for both the wild type and mutated complex. The average occupancy (rate of the frames with inter-residue distance within the 0.6 nm threshold), normalized ratio of contacts formation/breaking and mean lifetime (in ps units) are reported in columns 2 and 5, 3 and 6, 4 and 7, respectively.

		c728 TCR HLA-A*02:01/MAGE-A10			c796 TCR HLA-A*02:01/MAGE-A10	
	occupancy	formation / breaking	lifetime	occupancy	formation / breaking	lifetime
αCDR1-R29 – E59	0.175	0.024	57.533	0.346	0.036	82.986
αCDR3-R98 – pD4	0.599	0.067	203.165	0.111	0.022	41.688
βCDR3-D98 – H152	0.525	0.034	199.158	1.000	0.000	200695.930
βCDR2-E53 – R76	0.146	0.027	50.968	0.408	0.006	758.992
βCDR2-E53 – R83	0.122	0.030	29.658	0.323	0.023	272.162
βCDR1-H30 – pH8	0.714	0.104	170.543	0.853	0.043	34741.839
βCDR1-H30 – K147	0.587	0.082	162.293	0.982	0.008	1670.401
βCDR2-K57 – E155	0.372	0.075	47.550	0.907	0.032	424.658

TABLE S4 Preferential allosteric pathways. The total weights of the preferential allosteric pathways, listed in the first column, are shown in the third column. The second column reports the individual weights representing the correlations between neighboring residues.

System / Pathway	Allosteric Pathway	Weight
c728 TCR / βE31 $\rightarrow$ βH30	βE31 $\xrightarrow{0.055}$ βH30	0.055
c796 TCR / βD31 $\rightarrow$ βH30	βD31 $\xrightarrow{0.062}$ βH30	0.062
c728 TCR / βE31 $\rightarrow$ βE53	βE31 $\xrightarrow{0.123}$ βG52 $\xrightarrow{0.069}$ βE53	0.192
c796 TCR / βD31 $\rightarrow$ βE53	βD31 $\xrightarrow{0.120}$ βG52 $\xrightarrow{0.079}$ βE53	0.199
c728 TCR / βE31 $\rightarrow$ βD98	βE31 $\xrightarrow{0.083}$ βS95 $\xrightarrow{0.084}$ βT97 $\xrightarrow{0.065}$ βD98	0.231
c796 TCR / βD31 $\rightarrow$ βD98	βD31 $\xrightarrow{0.062}$ βH30 $\xrightarrow{0.113}$ βF96 $\xrightarrow{0.096}$ βD98	0.271
c728 TCR / βE31 $\rightarrow$ αR29	βE31 $\xrightarrow{0.061}$ βY32 $\xrightarrow{0.130}$ αR98 $\xrightarrow{0.098}$ αR93 $\xrightarrow{0.079}$ αS31 $\xrightarrow{0.091}$ αR29	0.458
c796 TCR / βD31 $\rightarrow$ αR29	βD31 $\xrightarrow{0.067}$ βY32 $\xrightarrow{0.168}$ αR98 $\xrightarrow{0.109}$ αR93 $\xrightarrow{0.084}$ αS31 $\xrightarrow{0.083}$ αR29	0.511
c728 TCR / βE31 $\rightarrow$ αK57	βE31 $\xrightarrow{0.083}$ βS95 $\xrightarrow{0.097}$ βT99 $\xrightarrow{0.116}$ αF35 $\xrightarrow{0.062}$ αS50 $\xrightarrow{0.099}$ αK57	0.456
c796 TCR / βD31 $\rightarrow$ αK57	βD31 $\xrightarrow{0.067}$ βY32 $\xrightarrow{0.078}$ βS95 $\xrightarrow{0.099}$ βT99 $\xrightarrow{0.140}$ αL47 $\xrightarrow{0.068}$ αM49 $\xrightarrow{0.121}$ αK57	0.573
c728 TCR / βE31 $\rightarrow$ αR98	βE31 $\xrightarrow{0.061}$ βY32 $\xrightarrow{0.130}$ αR98	0.191
c796 TCR / βD31 $\rightarrow$ αR98	βD31 $\xrightarrow{0.067}$ βY32 $\xrightarrow{0.168}$ αR98	0.235

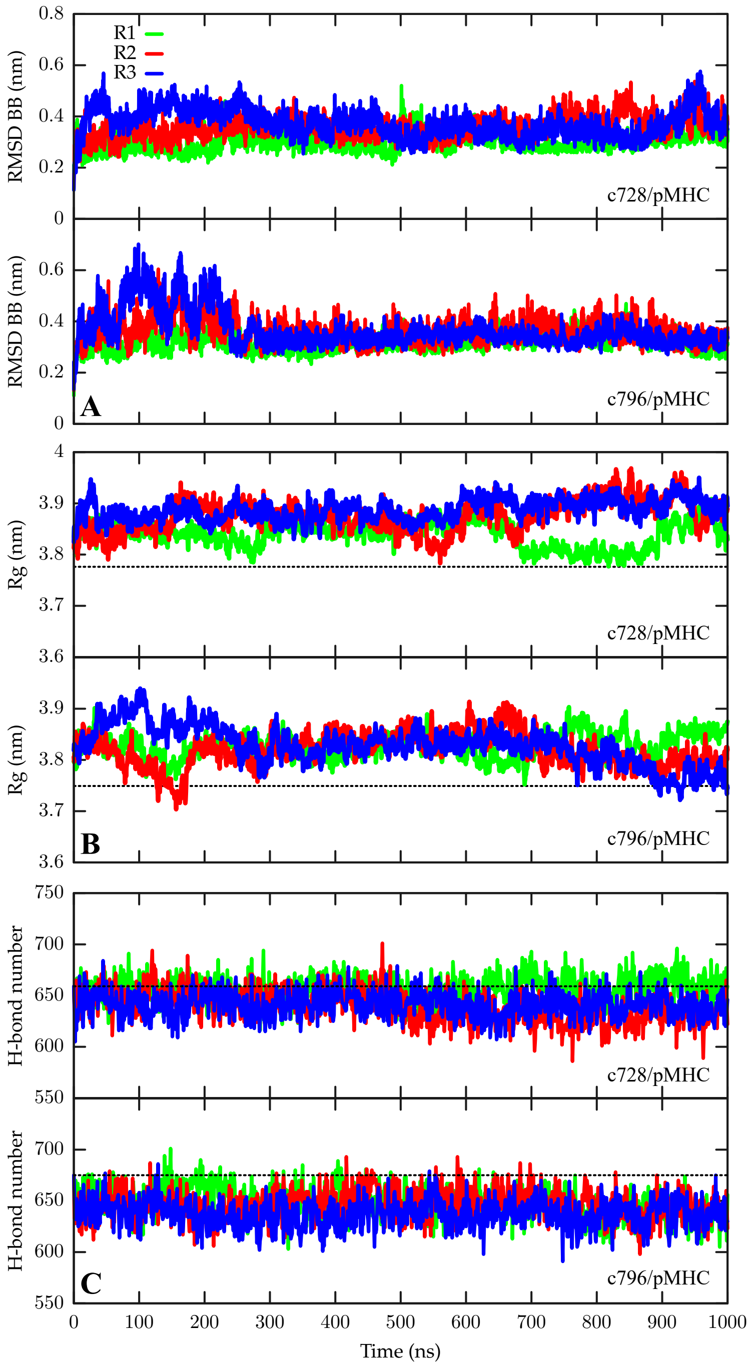

FIGURE S1 Overall structural stability of the two simulated TCR-pMHC complexes. A) RMSD calculated for the protein backbone of each simulated TCR-pMHC complex relative to the starting structure, plotted as a function of time. B) Radius of gyration of each simulated TCR-pMHC complex over time. The dotted line indicates the value corresponding to the X-ray structure. C) Number of H-bonds present in the entire complex as a function of time for each simulated TCR-pMHC complex. The dotted line indicates the value corresponding to the X-ray structure.

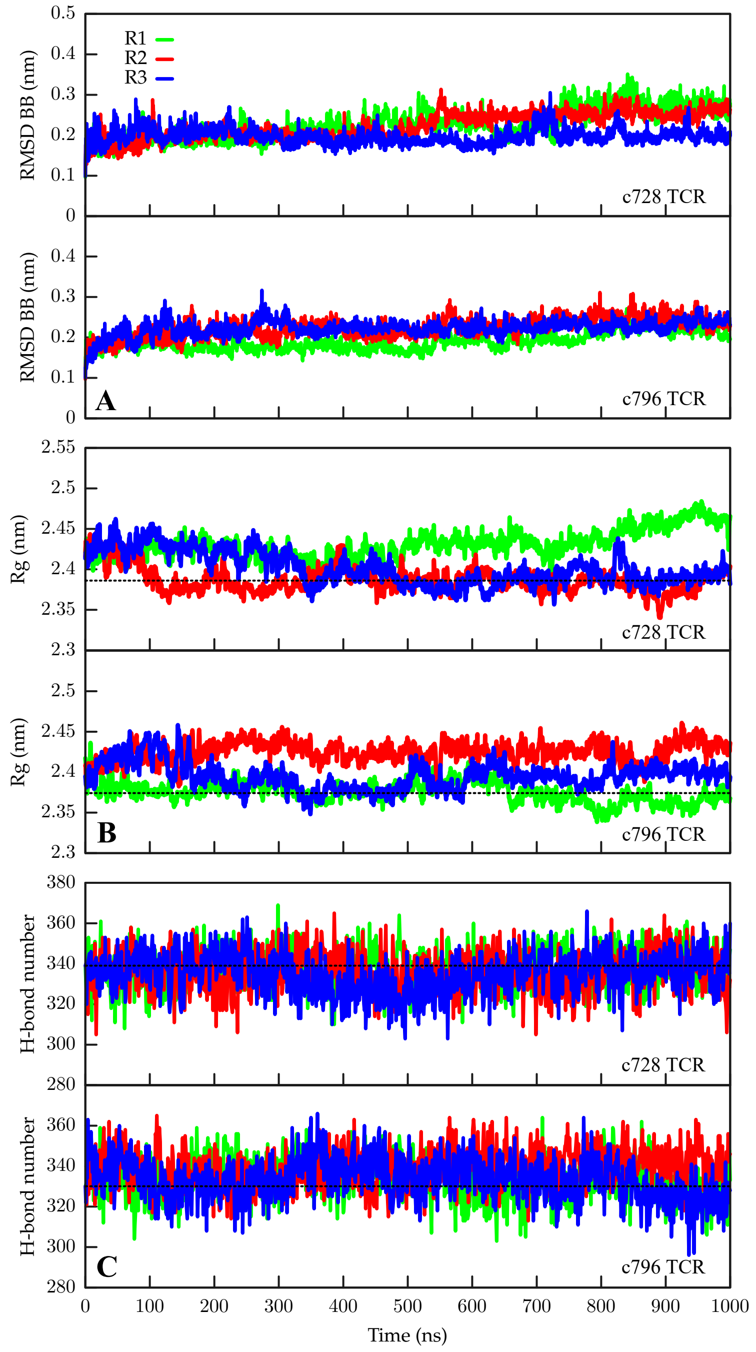

FIGURE S2 Overall structural stability of the two TCRs simulated in isolation. A) RMSD calculated for the protein backbone of each simulated TCR (c728 and c796) relative to the starting structure, plotted as a function of time. B) Radius of gyration of each simulated TCR as a function of time. The dotted line indicates the value corresponding to the X-ray structure. C) Number of H-bonds present in the protein as a function of time for each simulated TCR. The dotted line indicates the value corresponding to the X-ray structure.

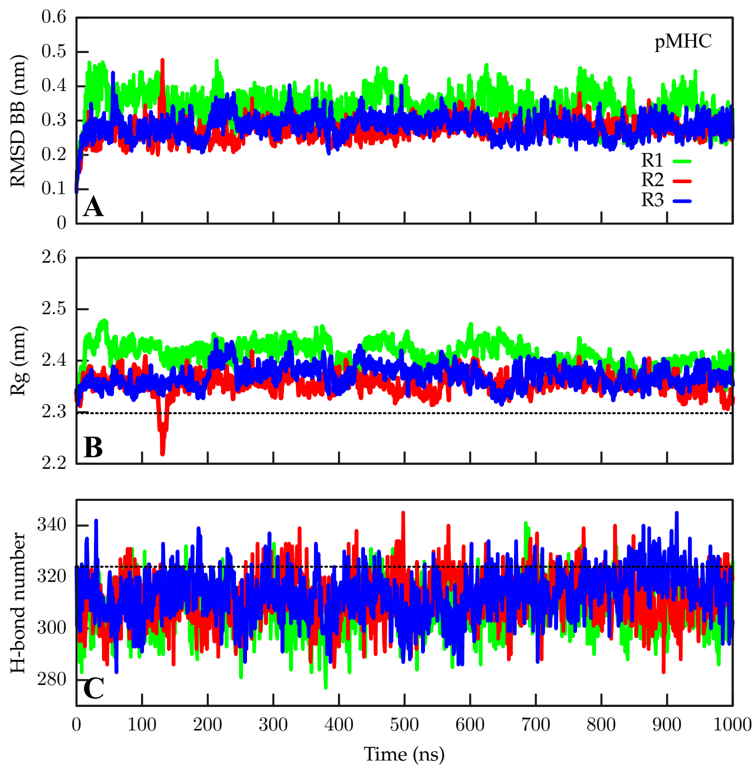

FIGURE S3 Overall structural stability of the HLA-A*02:01/MAGE-A10 complex simulated in isolation. A) RMSD of the pMHC backbone relative to the starting structure, plotted as a function of time. B) Radius of gyration of the pMHC as a function of time. The dotted line indicates the value corresponding to the X-ray structure. C) Number of H-bonds in the pMHC as a function of time. The dotted line indicates the value corresponding to the X-ray structure.

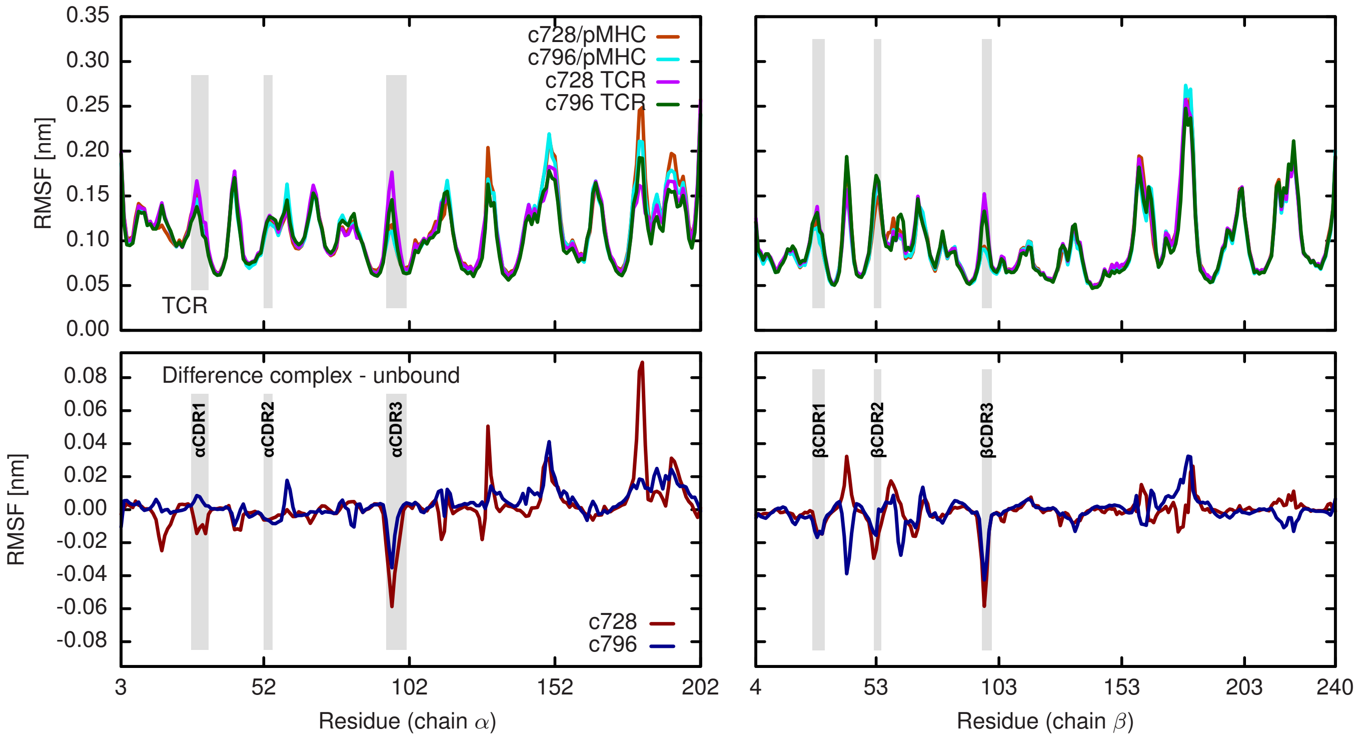

FIGURE S4 RMSF of the amino acid backbone in chain α (left) and chain β (right) of the c728 TCR (orange trace) and c796 TCR (cyan trace) simulated in complex with HLA-A*02:01/MAGE-A10, and of the c728 TCR (magenta trace) and c796 TCR (green trace) simulated in isolation, calculated over their respective trajectories. The difference in RMSF between each TCR simulated bound to HLA-A*02:01/MAGE-A10 and in isolation is shown in the bottom panels as a red trace (c728) and a blue trace (c796). TCR loops interacting with the HLA-A*02:01/MAGE-A10 complex are highlighted in gray. All RMSF values were block-averaged over 100 ns time windows, excluding the first 200 ns of each trajectory.

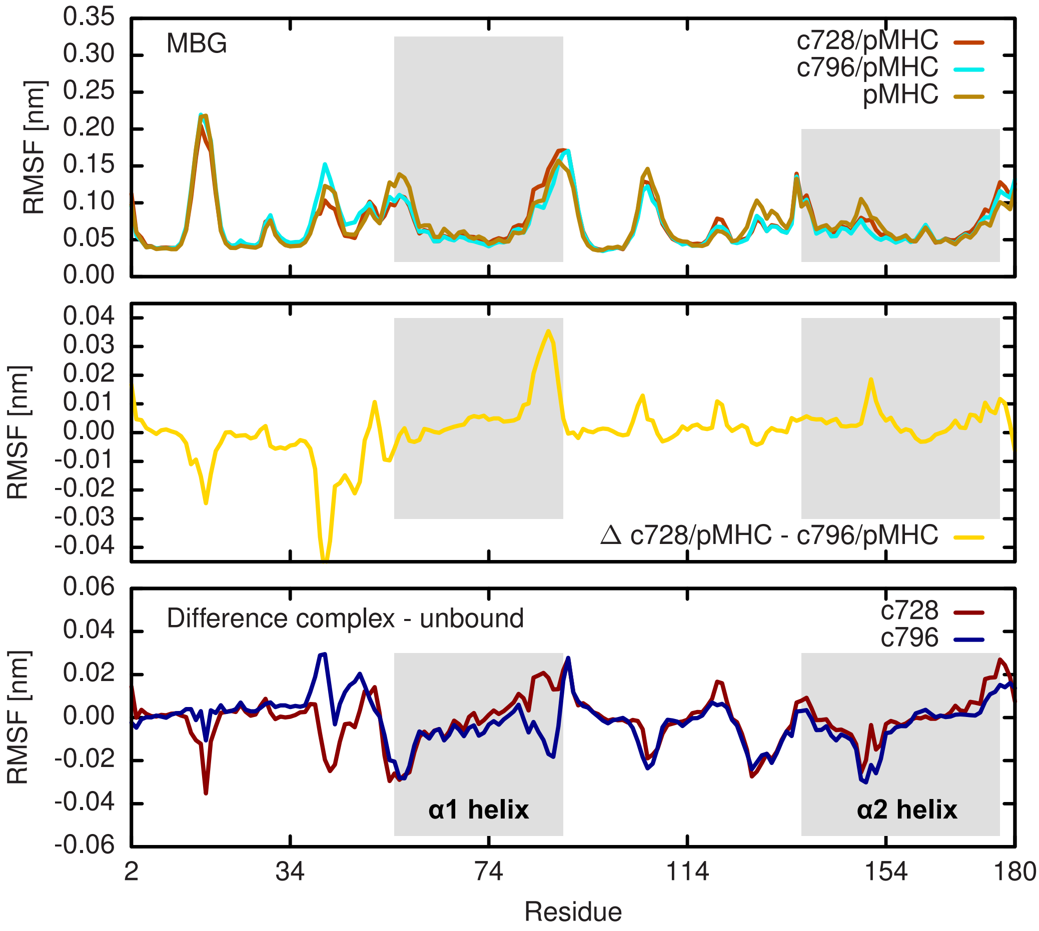

FIGURE S5 Top panel: RMSF of the amino acid backbone of the binding groove of HLA-A*02:01 in complex with the c728 TCR (red trace), the c796 TCR (cyan trace), and in isolation (brown trace). Middle panel: the difference in RMSF between HLA-A*02:01 bound to the c728 TCR and HLA-A*02:01 bound to the c796 TCR is shown as a yellow trace. Bottom panel: the difference in RMSF between HLA-A*02:01 bound to a TCR and HLA-A*02:01 simulated in isolation is shown as a dark red trace (pMHC bound to c728 TCR) and a blue trace (pMHC bound to c796 TCR). The $\alpha 1$ and $\alpha 2$ helices lining the binding groove are highlighted in gray. All RMSF values were block-averaged over 100 ns time windows, excluding the first 200 ns of each trajectory.

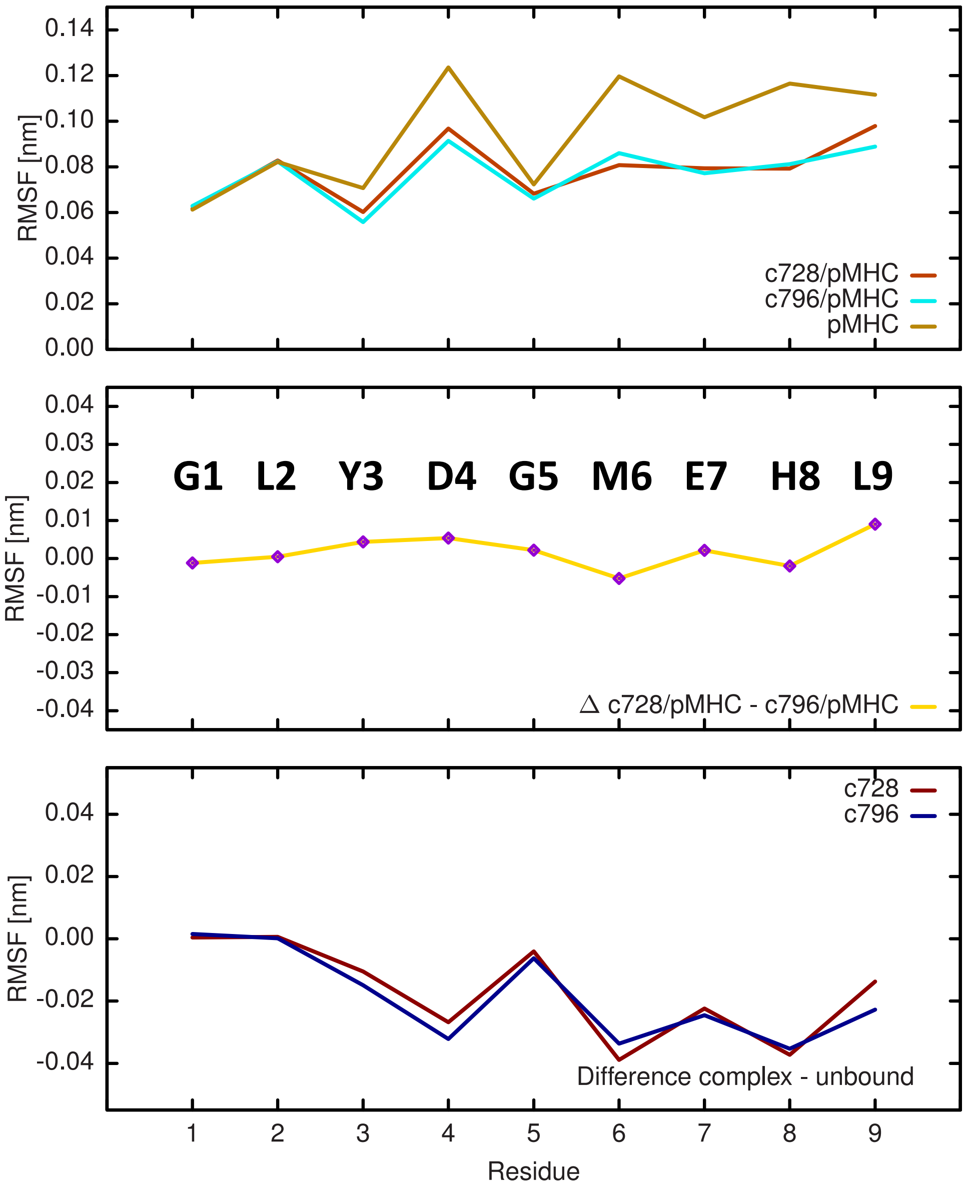

FIGURE S6 RMSF of the amino acid side chains of the MAGE-A10 peptide bound to HLA-A*02:01 in complex with the c728 TCR (orange trace), the c796 TCR (cyan trace), and bound only to HLA-A*02:01 (brown trace). The difference in RMSF between MAGE-A10 bound to HLA-A*02:01 in complex with the c728 TCR and with the c796 TCR is shown in the middle panel as a yellow line. The difference in RMSF between MAGE-A10 peptide bound to HLA-A*02:01 in complex with TCRs and bound only to HLA-A*02:01 is shown in the bottom panel as a red trace (pMHC bound to c728 TCR) and a blue trace (pMHC bound to c796 TCR). All RMSF values were block-averaged over 100 ns time windows, excluding the first 200 ns of each trajectory.

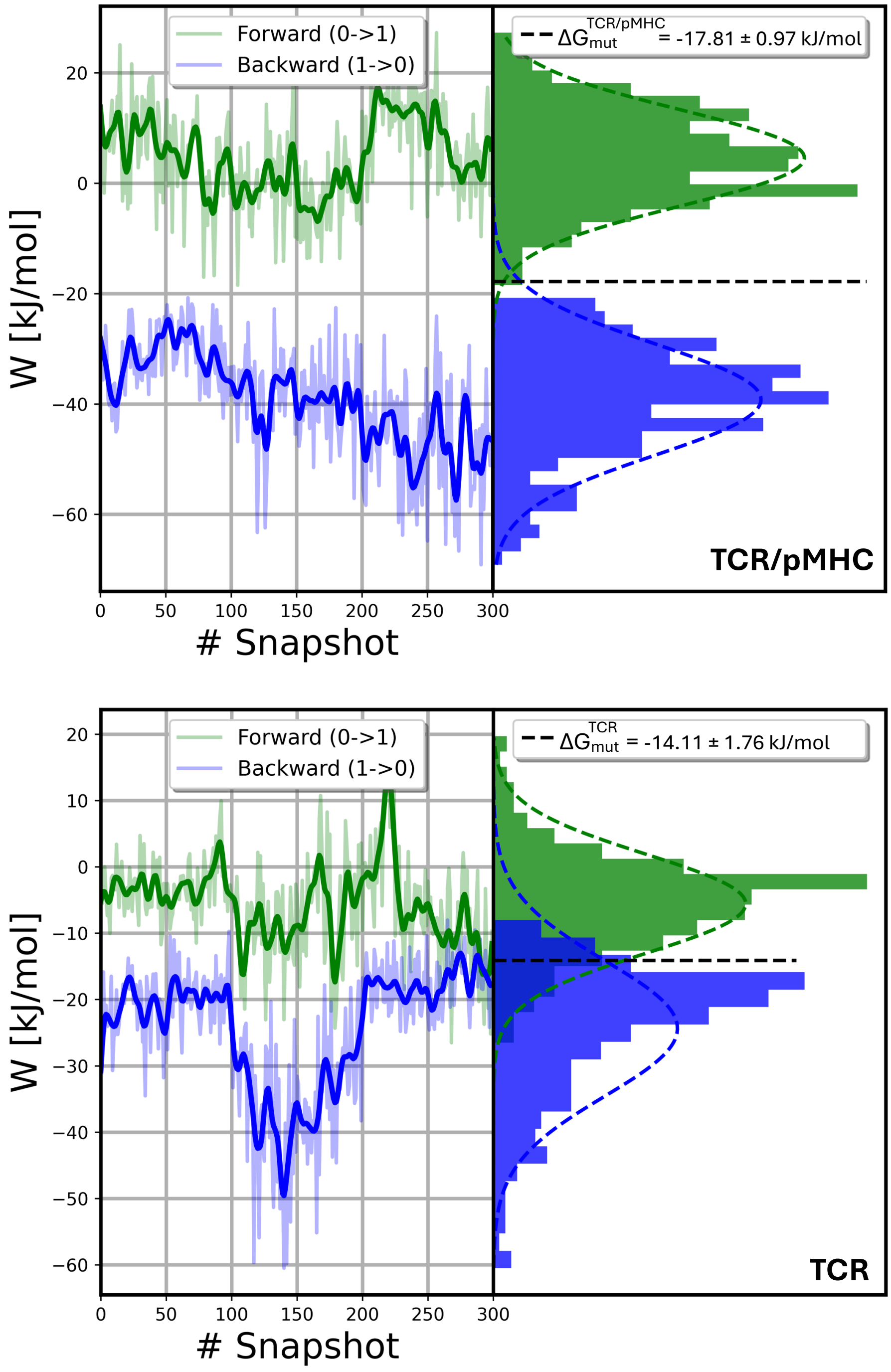

FIGURE S7 Free Energy Perturbation analysis. Outputs generated by the pmx analyze script for the TCR/pMHC complex (top panel) and the isolated TCR (bottom panel). On the left, the work values for the forward and reverse transitions are shown for each frame extracted from the equilibrium MD trajectories. On the right, histograms report the distributions of these work values, whose overlap allows the estimation of the free energy difference. In our case, the BAR estimator yielded a ΔG value of -17.81 ± 0.97 kJ/mol for the E31D mutation in the TCR/pMHC complex, and a ΔG value of -14.11 ± 1.76 kJ/mol for the same mutation in the isolated TCR.

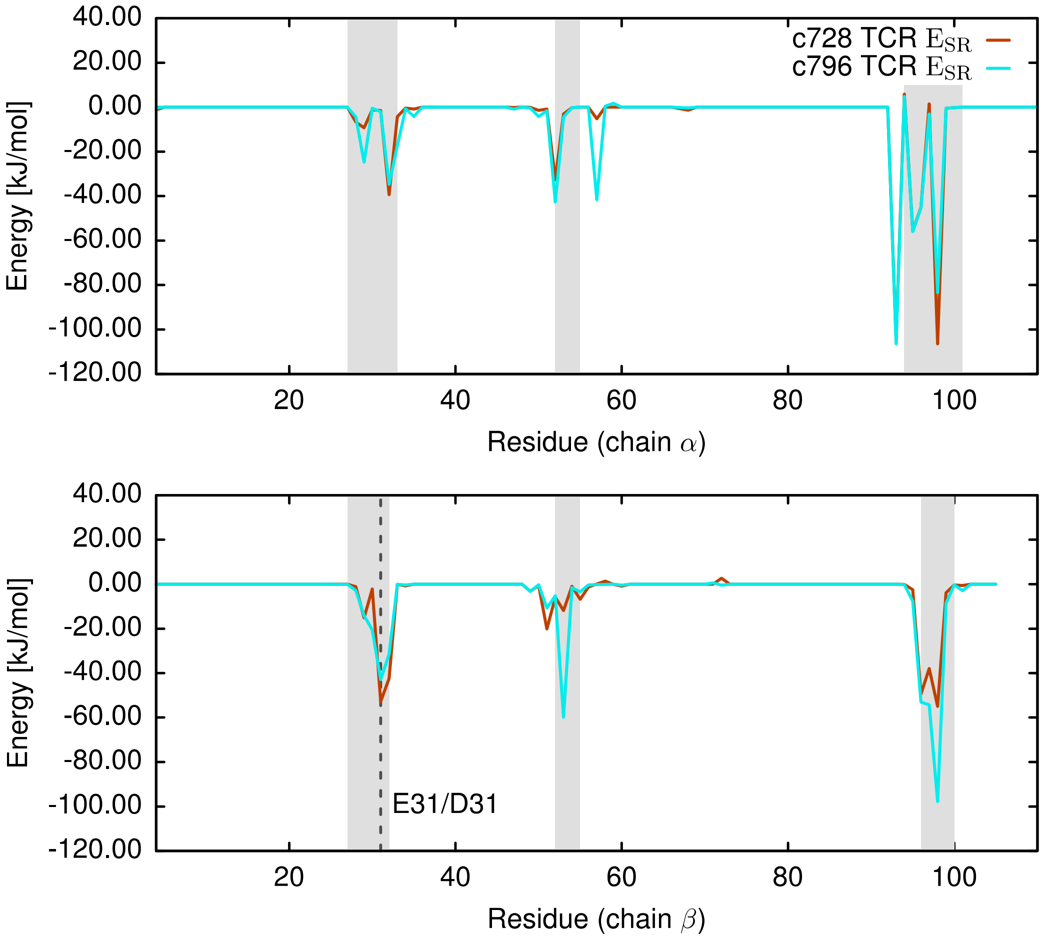

FIGURE S8 Interaction energy of TCR residues with pMHC. Interaction energies (ΔE_{SR}) for selected residues of the α chain of the TCRs with pMHC are shown as an orange trace (c728 TCR) and a cyan trace (c796 TCR) in the top panel. Interaction energies (ΔE_{SR}) for selected residues of the β chain of the TCRs with pMHC are shown as an orange trace (c728 TCR) and a cyan trace (c796 TCR) in the bottom panel. TCR loops interacting with the HLA-A*02:01/MAGE-A10 complex are highlighted in gray.

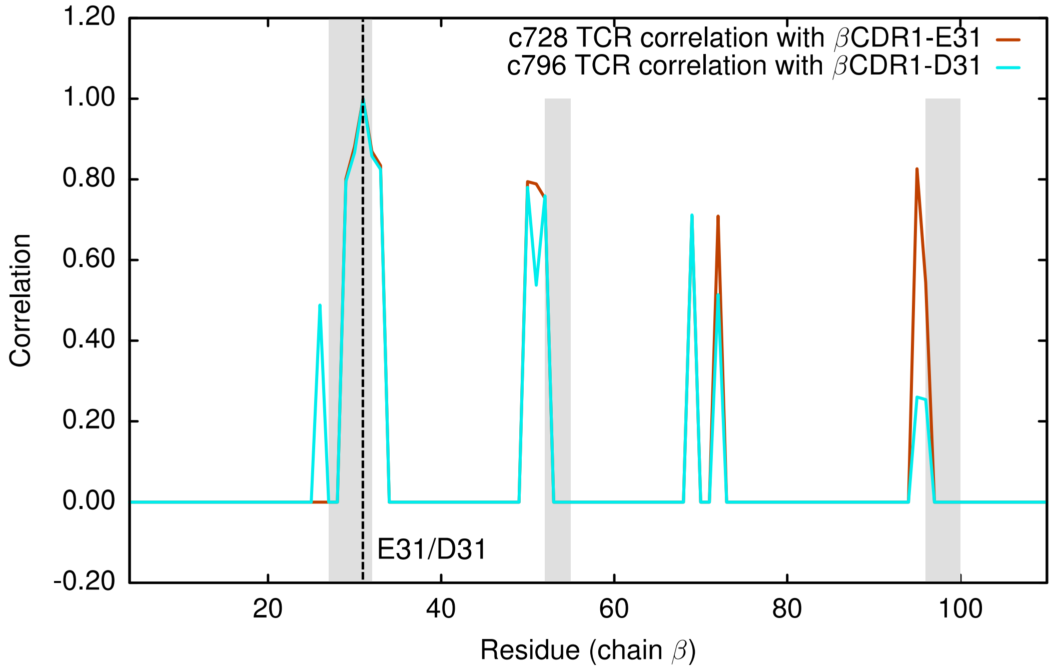

FIGURE S9 Correlations between the βCDR1-E31 residue and residues in the β chain of the c728 TCR are shown as an orange trace. Correlations between the βCDR1-D31 residue and residues in the β chain of the c796 TCR are shown as a cyan trace. TCR loops interacting with the HLA-A*02:01/MAGE-A10 complex are highlighted in gray.
